# A Cyclic Metabolic Network in Pseudomonas protegens Pf-5 Prioritizes the Entner-Doudoroff Pathway and Exhibits Substrate Hierarchy during Carbohydrate Co-Utilization

**DOI:** 10.1128/AEM.02084-18

**Published:** 2018-12-13

**Authors:** Rebecca A. Wilkes, Caroll M. Mendonca, Ludmilla Aristilde

**Affiliations:** a Department of Biological and Environmental Engineering, College of Agriculture and Life Sciences, Cornell University, Ithaca, New York, USA; Goethe University Frankfurt am Main

**Keywords:** *Pseudomonas*, carbohydrate co-utilization, hexose sugar, metabolic flux analysis, metabolomics

## Abstract

The genetic characterization of Pseudomonas protegens Pf-5 was recently completed. However, the inferred metabolic network structure has not yet been evaluated experimentally. Here, we employed ^13^C-tracers and quantitative flux analysis to investigate the intracellular network for carbohydrate metabolism. In lieu of the direct phosphorylation of glucose by glucose kinase, glucose catabolism was characterized primarily by the oxidation of glucose to gluconate and 2-ketogluconate before the phosphorylation of these metabolites to feed the Entner-Doudoroff (ED) pathway. In the absence of phosphofructokinase activity, a cyclic flux from the ED pathway to the upper Embden-Meyerhof-Parnas (EMP) pathway was responsible for routing glucose-derived carbons to the non-oxidative pentose phosphate (PP) pathway. Consistent with the lack of annotated genes in P. protegens Pf-5 for the transport or initial catabolism of pentoses and galactose, only glucose was assimilated into intracellular metabolites in the presence of xylose, arabinose, or galactose. However, when glucose was fed simultaneously with fructose or mannose, co-uptake of these hexoses was evident, but glucose was preferred over fructose (3 to 1) and over mannose (4 to 1). Despite gene annotation of mannose catabolism to fructose-6-phosphate, metabolite labeling patterns revealed that mannose was assimilated into fructose-1,6-bisphosphate, similarly to fructose catabolism. Remarkably, carbons from mannose and fructose were also found to cycle backward through the upper EMP pathway toward the ED pathway. Therefore, the operational metabolic network for processing carbohydrates in P. protegens Pf-5 prioritizes flux through the ED pathway to channel carbons to EMP, PP, and downstream pathways.

**IMPORTANCE** Species of the *Pseudomonas* genus thrive in various nutritional environments and have strong biocatalytic potential due to their diverse metabolic capabilities. Carbohydrate substrates are ubiquitous both in environmental matrices and in feedstocks for engineered bioconversion. Here, we investigated the metabolic network for carbohydrate metabolism in Pseudomonas protegens Pf-5. Metabolic flux quantitation revealed the relative involvement of different catabolic routes in channeling carbohydrate carbons through a cyclic metabolic network. We also uncovered that mannose catabolism was similar to fructose catabolism, despite the annotation of a different pathway in the genome. Elucidation of the constitutive metabolic network in P. protegens is important for understanding its innate carbohydrate processing, thus laying the foundation for targeting metabolic engineering of this untapped *Pseudomonas* species.

## Abstract (precis)

Species of the *Pseudomonas* genus thrive in various nutritional environments and have strong biocatalytic potential due to their diverse metabolic capabilities. Carbohydrate substrates are ubiquitous both in environmental matrices and in feedstocks for engineered bioconversion. Here, we investigated the metabolic network for carbohydrate metabolism in Pseudomonas protegens Pf-5. Metabolic flux quantitation revealed the relative involvement of different catabolic routes in channeling carbohydrate carbons through a cyclic metabolic network. We also uncovered that mannose catabolism was similar to fructose catabolism, despite the annotation of a different pathway in the genome. Elucidation of the constitutive metabolic network in P. protegens is important for understanding its innate carbohydrate processing, thus laying the foundation for targeting metabolic engineering of this untapped *Pseudomonas* species.

## INTRODUCTION

Species of the genus *Pseudomonas*, which are ubiquitous in the environment, are metabolically diverse and often touted for industrial bioproduction (1). The elucidation of the cellular network of carbon fluxes through metabolic pathways of these bacterial species is critical both to understand their role in carbon processing in environmental matrices and to engineer these species to optimize their use in agriculture, industry, and medicine. Gaining importance in bioremediation, Pseudomonas protegens Pf-5 was identified to produce enzymes that degrade polyurethane, a plastic polymer (2). Furthermore, P. protegens Pf-5 is also known to synthesize and release several antimicrobials and exoenzymes that are toxic to plant pathogens (3–6). Recently, P. protegens Pf-5 was characterized and annotated at the genomic level (7). However, the metabolic network of P. protegens Pf-5 has only been inferred from the genome annotation and has not yet been investigated experimentally.

Given the importance and ubiquity of carbohydrate-containing feedstocks, we sought to unravel the metabolic network structure for carbohydrate metabolism in P. protegens Pf-5 by combining ^13^C-assisted cellular carbon mapping with ^13^C metabolic flux analysis (MFA). Previous studies on other *Pseudomonas* species (i.e., P. putida and P. fluorescens) focused on elucidating the metabolic fluxes during growth on glucose, a prototypical carbohydrate substrate (8–11). In a similar fashion, we also studied the innate carbohydrate metabolism in P. protegens Pf-5 during feeding on glucose alone. However, carbon feedstocks are typically composed of other carbohydrates in addition to glucose. Therefore, we also investigated carbon assimilation and fluxes when the P. protegens Pf-5 cells were fed on mixtures of glucose with other hexoses (mannose, fructose, and galactose) or pentoses (xylose and arabinose).

**FIG 1:**
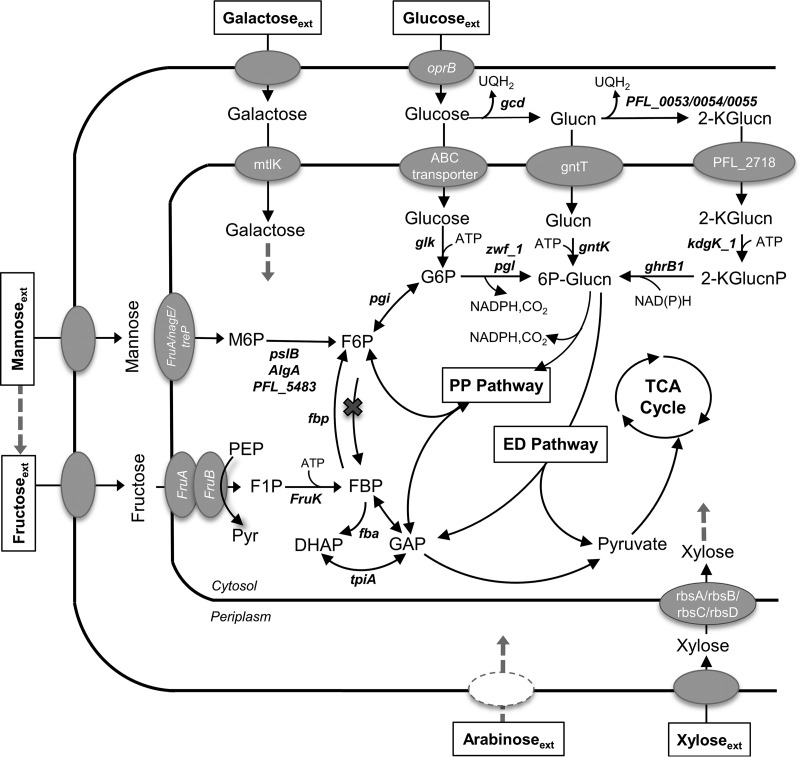
Putative genes involved in the uptake and initial catabolism of glucose, galactose, mannose, fructose, xylose, and arabinose into central metabolism. The gene annotations for pathways were collected from KEGG database (37–39) and MetaCyc database (40) for P. protegens Pf-5. The corresponding gene loci for the genes in the figure are shown in Table S1 in the supplemental material. Glucn, gluconate; 2-KGlucn, 2-ketogluconate; 2-KGlucnP, 2-keto-6-phosphogluconate; G6P, glucose 6-phosphate; 6P-Glucn, 6-phosphogluconate; F6P, fructose 6-phosphate; FBP, fructose 1,6-bisphosphate; DHAP, dihydroxyacetone-3-phosphate; GAP, glyceraldehyde 3-phosphate; F1P, fructose 1-phosphate; Pyr, pyruvate; PEP, phosphoenolpyruvate.

Previous reports showed that P. protegens strains were able to grow on glucose, mannose, or fructose as a single carbon source but not on galactose, xylose, or arabinose (6). In the genome of P. protegens Pf-5, genes for the following transporters were found: a phosphoenolpyruvate (PEP)/sugar phosphotransferase system (PTS) for fructose uptake (and possibly for mannose uptake) and an ATP-binding cassette (ABC) transporters for glucose, mannose, galactose, and xylose (Fig. 1; see also Table S1 in the supplemental material) (7). Characteristically in *Pseudomonas* species, glucose metabolism involves a peripheral pathway in the periplasm wherein glucose dehydrogenase converts glucose to gluconate and gluconate 2-dehydrogenase converts gluconate to 2-ketogluconate (Fig. 1) (8). Fluxes through these oxidation reactions were found to maximize growth on glucose (8). After active transport into the cytoplasm, the oxidized products of glucose are phosphorylated to 6-phosphogluconate (6P-gluconate) and subsequently routed to the Entner-Doudoroff (ED) pathway or the pentose phosphate (PP) pathway (Fig. 1) (8, 10, 11). Previous studies with other *Pseudomonas* species have determined that the forward Embden-Meyerhof-Parnas (EMP) pathway was not possible due to the absence of 6-phosphofructokinase to convert fructose-6-phosphate (F6P) to fructose-1,6-bisphosphate (FBP) (Fig. 1) (1, 7, 10). Therefore, to route glucose-derived carbons eventually toward biosynthetic pathways and the tricarboxylic acid (TCA) cycle, the ED pathway is required, wherein 6P-gluconate is cleaved to produce pyruvate and glyceraldehyde 3-phosphate (GAP) (Fig. 1). The gene that encodes 6-phosphofructokinase was also found to be absent in P. protegens Pf-5 (7); the ED pathway is thus assumed to be the required route for glucose metabolism in P. protegens Pf-5. However, the extent to which the peripheral pathway of glucose oxidation contributes to the initial glucose catabolism compared to direct glucose phosphorylation to glucose-6-phosphate (G6P) in P. protegens Pf-5 remains to be determined.

In contrast to glucose, fructose is transported through the PTS, which uses the phosphate group from PEP to phosphorylate fructose to fructose-1-phosphate (F1P) followed by a subsequent phosphorylation step by 1-phosphofructokinase to convert F1P to FBP (Fig. 1) (9, 12, 13). Previous studies on other *Pseudomonas* species (P. fluorescens, P. putida, P. aeruginosa, P. stutzeri, and P. acidovorans) found that fructose-derived carbons were cycled backward via a reverse (gluconeogenic) flux through the upper EMP pathway (FBP up to G6P) to be connected to the ED pathway (12, 14–16). However, according to the genome of P. protegens Pf-5, FBP could also be involved in a forward flux of the EMP pathway, wherein FBP is lysed directly to glyceraldehyde-3-phosphate (GAP) and dihydroxyacetone-3-phosphate (DHAP) (Fig. 1) (7). Whether the preferential route for fructose assimilation during growth on mixed carbohydrates is via the direct route of FBP to the triose phosphates or through the reverse cycling of carbons from FBP to the ED pathway remains to be determined. The catabolic routing of FBP has important energetic implications for P. protegens Pf-5. Compared to the direct FBP lysis through the forward EMP pathway, the combination of reverse flux through the upper EMP pathway with the ED pathway maintains the same quantity of reduced equivalents [i.e., NAD(P)H] but half the ATP yield.

With respect to initial mannose catabolism, the authors of previous studies with P. aeruginosa proposed two possible routes, which involve either mannose isomerization to fructose followed by subsequent phosphorylation to FBP or direct phosphorylation of mannose to mannose-6-phosphate (M6P) prior to isomerization to F6P (17, 18). Relevant to the first route, an intracellular mannose isomerase (EC 5.3.1.7) was reported in P. cepacia and P. saccharophila (19, 20), but the gene for this enzyme was not annotated in the P. protegens Pf-5 genome (7). On the other hand, albeit not yet confirmed by metabolic studies, the genes for the relevant enzymes in the second catabolic route, i.e., the conversion of mannose to F6P, were annotated in the P. protegens Pf-5 genome (Fig. 1).

Both oxidative and non-oxidative routes of the PP pathway are important to channel carbohydrate-derived metabolite precursors to the biosynthesis of ribonucleotides and aromatic amino acids. The presence of the annotated genes for transketolase and transaldolase enzymes implied a fully functional PP pathway in P. protegens Pf-5 (7). For xylose catabolism, the annotation of a ribose transporter indicates that it could be used as a possible xylose transporter, but the genes encoding the enzymes (xylose isomerase and xylulose kinase) responsible for introducing xylose into the PP pathway were not present in P. protegens Pf-5 (Fig. 1) (7). Moreover, the collective enzymes needed for the alternative route for xylose through the Weimberg pathway, which incorporates xylose through xylonate into α-ketoglutarate, were not all present in the genome of P. protegens Pf-5 (7). Regarding arabinose catabolism, there was no annotated pathway for the assimilation of arabinose in P. protegens Pf-5 (Fig. 1) (7). Despite the lack of the relevant genes for xylose catabolism, a recent study found extracellular xylose depletion by P. protegens Pf-5 during growth on a mixture of carbohydrates (21). Whether arabinose or xylose is incorporated into the PP pathway by some other non-annotated pathway in the presence of another carbohydrate remains to be determined.

Here we applied liquid chromatography (LC) with high-resolution mass spectrometry (HRMS) to perform a ^13^C-assisted metabolomics investigation during growth of P. protegens Pf-5 on glucose alone or simultaneously with fructose, mannose, galactose, xylose, or arabinose. We provide the first quantitative evaluation of the hypothetical metabolic network of P. protegens Pf-5 deduced from its genome-level characterization. First, we employed carbon mapping to identify the specific pathways that channel glucose-derived carbons throughout cellular metabolism. Second, we performed quantitative analyses to determine the carbon fluxes and energetic yields in the cellular metabolism in P. protegens Pf-5. Third, we determined which carbohydrates can be co-assimilated with glucose in P. protegens Pf-5 and subsequently quantified the metabolic fluxes when co-utilization occurred. Our findings provide new metabolic insights, which both resolve discrepant metabolic predictions from genome annotation and quantify fluxes in the metabolic network structure for carbohydrate processing in P. protegens Pf-5.

## RESULTS

### Physiological parameters of P. protegens Pf-5 grown on carbohydrate mixtures.

**FIG 2:**
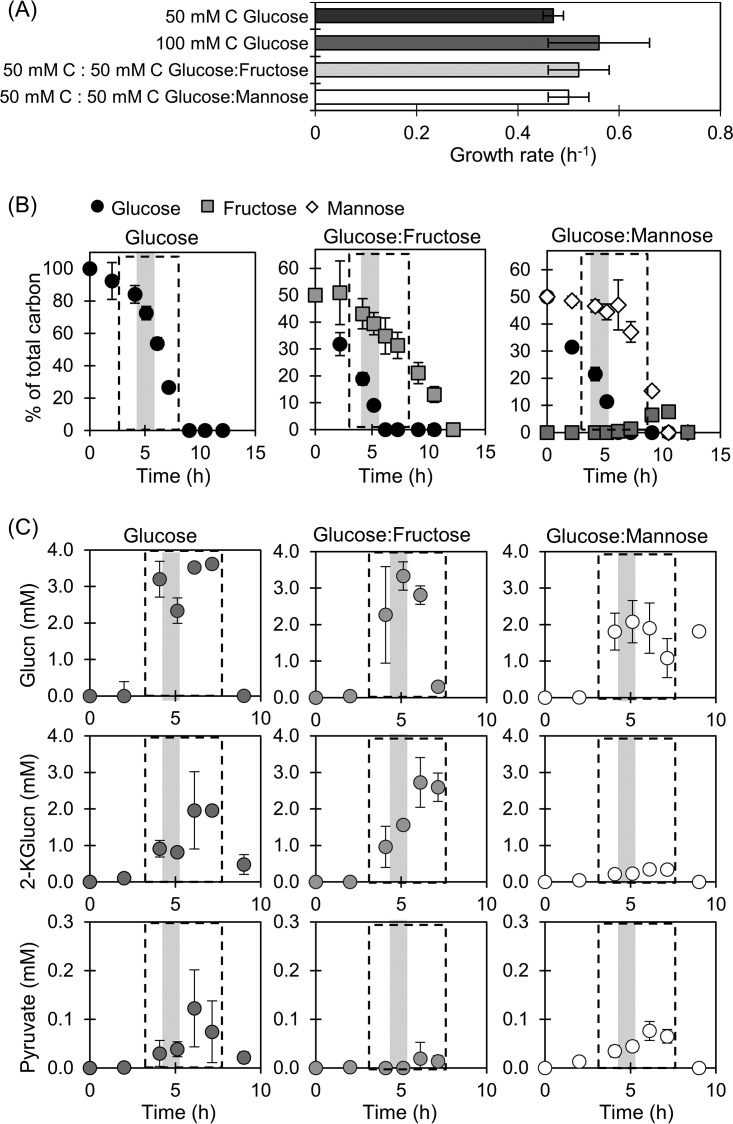
Growth rates (A), kinetic profiles of carbohydrate depletion (B), and kinetic profiles of metabolite secretions (C) for cells grown on glucose alone, on an equimolar mixture of glucose and fructose, or on a mixture of glucose and mannose. Initial total carbon-equivalent carbohydrate concentration was 100 mM C (or 3 g·liter^−1^ substrate concentration). In panels B and C, the dashed boxes designate when data points were obtained during exponential growth; the shaded gray areas at an OD_600_ of 0.5 correspond to the time points for the measured labeling data used in the MFA. Data (average ± standard deviation) are from independent biological replicates (*n* = 3).

We investigated the growth phenotype of P. protegens during batch culturing on different hexose combinations. Our growth rates were in close agreement with the reported values for glucose-grown P. putida (0.56 ± 0.02 h^−1^) and P. fluorescens (0.49 ± 0.03 h^−1^) (8, 22). Starting with a total carbon concentration of 100 mM in the growth medium, the growth rate of cells fed on glucose alone (0.56 ± 0.09 h^−1^) was similar to the growth rate of cells fed on a 1:1 mixture of glucose and fructose (0.52 ± 0.06 h^−1^) or glucose and mannose (0.50 ± 0.04 h^−1^) (Fig. 2A; see also Fig. S1 and Table S2 in the supplemental material). We also found that the growth rate remained relatively unchanged when the cells were grown on 50 mM C glucose alone (0.47 ± 0.02 h^−1^) (Fig. 2A). Therefore, P. protegens would not be subjected to carbon limitation during growth on carbohydrate mixtures if either fructose or mannose was not assimilated from the mixture with glucose (Fig. 2A).

We monitored substrate consumption by the P. protegens Pf-5 cells by tracking the depletion of the carbohydrates from the extracellular medium (Fig. 2B; Table S2). The glucose-grown cells depleted glucose completely over a 10-h period (Fig. 2B). Over the same time period, the cells grown on the mixture of glucose and mannose depleted both substrates completely, but cells grown on the mixture of glucose and fructose depleted glucose completely and approximately 70% of fructose (Fig. 2B). We also observed that, during the growth of P. protegens Pf-5 on the mixture of glucose and mannose, fructose appeared in the extracellular medium approximately 1 h after mannose consumption started (Fig. 2A). The appearance of extracellular fructose implied the presence of a mannose isomerase, which is responsible for converting mannose to fructose in other *Pseudomonas* species (19, 20) (Fig. 2B). During growth on both hexose mixtures, glucose was consumed faster and depleted by 6 h of growth, at which time approximately 30% of the fructose was consumed but only 10% of the mannose was depleted by the cells (Fig. 2B). Thus, during hexose co-utilization under both of our experimental conditions, glucose provided the major carbon source for cellular metabolism.

We also monitored the extracellular overflow of metabolic products, a phenomenon that is widely reported in *Pseudomonas* species (8, 11, 23). Both oxidized products of glucose (i.e., gluconate and 2-ketogluconate) and the organic acid pyruvate were found at appreciable levels (>1 µM) in the extracellular medium (Fig. 2C). Secretions of gluconate and 2-ketogluconate were reported previously with P. putida (8, 11) and P. fluorescens (23); pyruvate secretion was reported previously with P. fluorescens (23). We found that the level of these secreted metabolites from P. protegens Pf-5 was dependent on the substrate composition in the growth medium. The highest secretions of gluconate and 2-ketogluconate (greater than 2 mM) were measured in the medium when cells were grown on glucose alone or glucose with fructose. In contrast, during growth on the mixture glucose and mannose, the highest secretion of gluconate and 2-ketogluconate decreased substantially, by ∼40% and ∼85%, respectively (Fig. 2C; Table S2). Compared to gluconate and 2-ketogluconate, pyruvate was secreted in smaller amounts (micromolar range), with the highest secretion (0.13 ± 0.08 mM) obtained when cells were grown on glucose alone (Fig. 2C; Table S2).

### Metabolic pathways and fluxes in glucose-fed cells.

**FIG 3:**
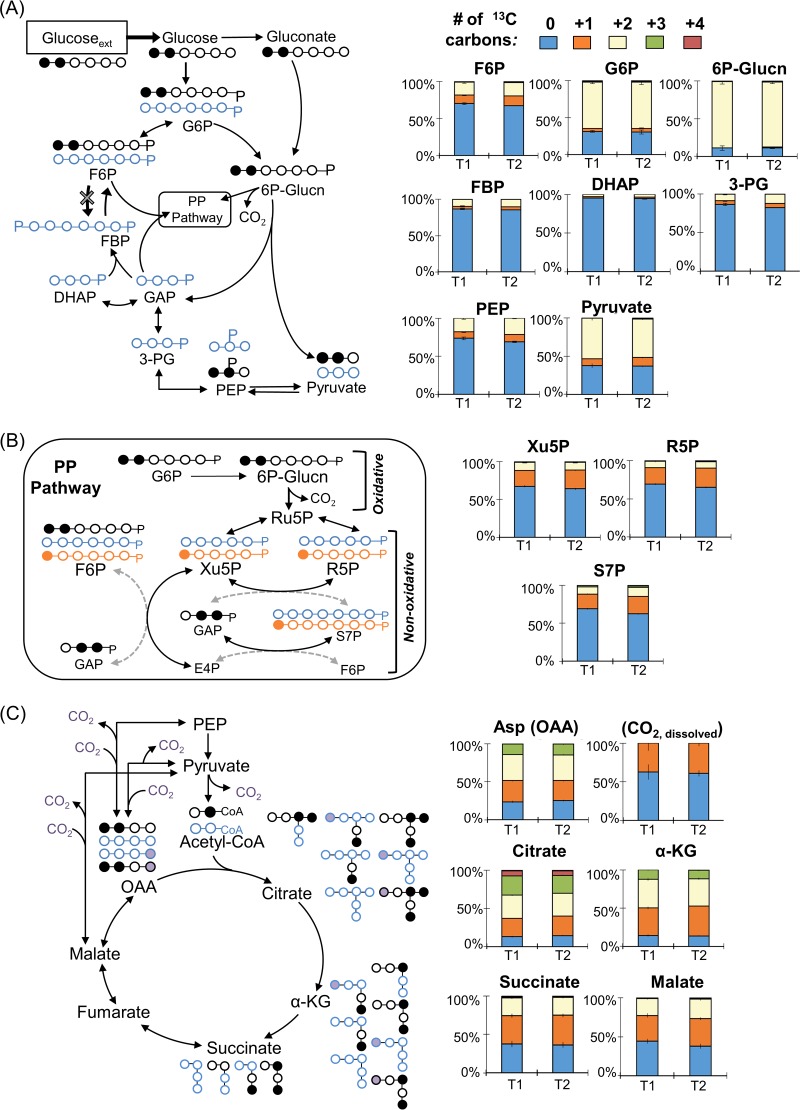
Long-term isotopic enrichment with [1,2-^13^C_2_]glucose for carbon mapping of the metabolic network structure in P. protegens Pf-5. Carbon mapping is illustrated on the left and the metabolite labeling data are provided on the right for the following: (A) Initial glucose catabolism, Embden-Meyerhof-Parnas (EMP) pathway, and the Entner-Doudoroff (ED) pathway; (B) oxidative and non-oxidative routes of the pentose-phosphate (PP) pathway; (C) the tricarboxylic acid (TCA) cycle. The dashed arrows describe minor formation routes of the metabolites. In the carbon mapping on the left, ^13^C carbons directly from glucose are in black, nonlabeled carbons from the ED pathway are in blue, singly ^3^C-labeled metabolites originated from the oxidative PP pathway are in orange, and the addition of labeled CO_2_ is shown by purple circles. Metabolite labeling patterns in panels A to C: nonlabeled (light blue), singly labeled (orange), doubly labeled (cream), triply labeled (green), and quadruply labeled (red). Labeling data (average ± standard deviation) were from independent biological replicates (*n =* 3). G6P, glucose 6-phosphate; 6P-Glucn, 6-phosphogluconate; F6P, fructose 6-phosphate; FBP, fructose 1,6-bisphosphate; DHAP, dihydroxyacetone-3-phosphate; GAP, glyceraldehyde 3-phosphate; PEP, phosphoenolpyruvate; 3-PG, 3-phosphoglycerate; Xu5P, xylulose 5-phosphate; R5P, ribose 5-phosphate; S7P, sedoheptulose 7-phosphate; OAA, oxaloacetate; Asp, aspartate; α-KG, α-ketogluconate.

**FIG 4:**
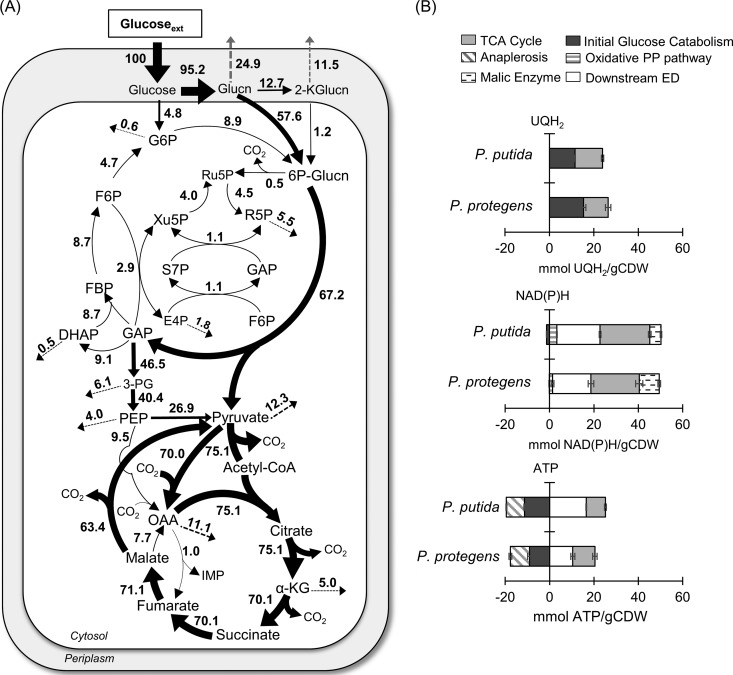
Quantitative metabolic flux analysis (A) and energetic distributions (B) in glucose-grown P. protegens Pf-5. All fluxes were normalized to glucose uptake, and the thickness of each arrow is scaled to the relative flux percentage. For panel A, black dotted arrows indicate contribution to biomass and gray dashed arrows indicate metabolite secretions. The absolute fluxes (average ± standard deviation), optimized on data obtained from independent biological replicates (*n =* 3), are listed in Table S3 and S4. Abbreviations in panel A are as listed in the legend of Fig. 3. For panel B, the relative contributions of different pathways to the NAD(P)H, UQH_2_, and ATP pools were calculated from metabolic fluxes. Data for P. putida KT2440 were obtained from the metabolic flux analysis reported in Nikel et al. (10). All values in panel B are relative to glucose consumption rate and cellular biomass.

Isotopic enrichment with [1,2-^13^C_2_]glucose was used to determine the metabolic network structure through the initial glucose catabolism, the EMP pathway, the ED pathway, the PP pathway, and the TCA cycle (Fig. 3). At two time points during exponential growth, we obtained similar metabolite ^13^C-labeling patterns, which confirmed pseudo-steady-state isotopic enrichment (Fig. 3). To elucidate the fluxes through 48 reactions in the metabolic pathway, we combined the metabolite labeling data with substrate consumption rates (accounting for the excretion of gluconate and 2-ketogluconate) and biomass growth rates (Fig. 4A; see also Fig. S2 and Tables S3 and S4). Adjusting for the carbon loss through metabolite secretions, approximately 64% of the glucose consumed from the extracellular medium was available for intracellular catabolism in P. protegens Pf-5. In conjunction with meeting the carbon mass balance constraints, the very close alignment between the MFA-estimated labeling patterns and those determined experimentally reflected the good quality of the optimization procedure for the model cellular fluxes (see Fig. S3).

**(i) Involvement of glucose oxidation and the ED pathway.** Glucose catabolism can be initiated via three different routes: direct phosphorylation to G6P, oxidation to gluconate in the periplasm before phosphorylation to 6P-gluconate, or additional periplasmic oxidation of gluconate to 2-ketogluconate before phosphorylation to 6P-gluconate (Fig. 1). Doubly ^13^C-labeled 6P-gluconate (86% to 89%) and doubly ^13^C-labeled G6P (∼63%) were both consistent with the assimilation of [1,2-^13^C_2_]glucose (Fig. 3A). In accordance with the ED pathway, wherein the first three carbons of 6P-gluconate become the doubly ^13^C-labeled pyruvate and the last three nonlabeled carbons become GAP, pyruvate was 50% to 53% doubly ^13^C-labeled, and DHAP (an isomer of GAP) was >95% nonlabeled (Fig. 3A). The 37% to 38% nonlabeled fraction of pyruvate was indicative of the nonlabeled fractions of precursor metabolites downstream of GAP (Fig. 3A). In the absence of the 6-phosphofructokinase enzyme in P. protegens Pf-5, and thus the lack of the traditional forward EMP pathway (7), the nonlabeled GAP and DHAP combined to produce the highly nonlabeled FBP (85% to 87%), which then cycled backward through the upper EMP pathway to result in the 67% to 70% nonlabeled F6P and 31% nonlabeled G6P (Fig. 3A). The MFA quantified the relative contribution of the three assimilation routes for the glucose-derived carbons in P. protegens Pf-5 (Fig. 4A). Only approximately 5% of the glucose uptake was directly converted to G6P in the cytosol, whereas up to 95% of glucose was oxidized to gluconate accompanied by another flux (12.7%) to 2-ketogluconate (Fig. 4A). Following the phosphorylation of the oxidized products of glucose to 6P-gluconate, our MFA determined that the flux through the ED pathway was 67.2% of the glucose uptake rate in P. protegens Pf-5 (Fig. 4A).

**(ii) Oxidative versus non-oxidative PP pathway.** Following a decarboxylation reaction through the oxidative PP pathway, doubly ^13^C-labeled 6P-gluconate would become singly ^13^C-labeled Ru5P, which would introduce singly ^13^C-labeled fractions into metabolites in the PP pathway (Fig. 3B). Thus, singly ^13^C-labeled fractions of xylulose 5-phosphate (Xu5P) (21% to 25%), ribose 5-phosphate (R5P) (22% to 25%), and sedoheptulose 7-phosphate (S7P) (20% to 23%) were due to flux through the oxidative PP pathway (Fig. 3B). However, by involving nonlabeled metabolites from downstream of the ED pathway, the non-oxidative PP pathway introduced relatively higher fractions of nonlabeled Xu5P (64% to 67%), R5P (65% to 69%), and S7P (62% to 69%) (Fig. 3B). Accordingly, the MFA determined an oxidative flux to the PP pathway (approximately 0.5%) that was a tenth of the non-oxidative PP pathway fluxes (5.1%) from ketolase and transaldolase reactions (Fig. 3B and 4A). As a precursor to both the oxidative PP pathway and the ED pathway, 6P-gluconate represents an important branch-point metabolite in the network. Therefore, the low contribution of 6P-gluconate to the oxidative PP pathway necessitated a cyclic flux from the downstream ED pathway to the upper EMP pathway to channel glucose-derived carbons toward the non-oxidative PP pathway to support biomass growth (Fig. 4A).

**(iii) Downstream metabolic pathways.** The ^13^C-labeling patterns of TCA cycle intermediates were consistent with the established route of carbon flow through this pathway (Fig. 3C). The decarboxylation of pyruvate generated nonlabeled and singly ^13^C-labeled acetyl moieties in acetyl coenzyme A (acetyl-CoA), which were subsequently incorporated into the TCA cycle by combining with oxaloacetate (OAA; nonlabeled, singly, doubly, and minorly triply ^13^C labeled) to produce citrate (nonlabeled, singly, doubly, triply, and minorly quadruply ^13^C labeled) (Fig. 3C). The two sequential decarboxylation reactions in the TCA cycle led to the disappearance of the quadruply ^13^C-labeled fraction in citrate and, thereafter, the triply ^13^C-labeled fraction in α-ketoglutarate (Fig. 3C). The resulting succinate labeling pattern (nonlabeled, singly, and doubly ^13^C labeled) led to a similar labeling scheme through fumarate, malate, and OAA (Fig. 3C). The MFA obtained a substantial flux (>70% of the glucose uptake) through the TCA cycle from OAA around to malate (Fig. 4A).

Anaplerotic reactions contributed to the triply ^13^C-labeled OAA (Fig. 3C). The aforementioned decarboxylation reactions in the TCA cycle contributed to the ^13^C-labeled carbon dioxide (CO_2_) pool, which was calculated to be ∼40% of the total dissolved CO_2_ (Fig. 3; see also Fig. S4). The carboxylation of doubly ^13^C-labeled pyruvate or doubly ^13^C-labeled PEP with ^13^C-labeled CO_2_ would generate triply ^13^C-labeled OAA (Fig. 3C). Notably, singly ^13^C-labeled OAA can be formed from carboxylation reactions of nonlabeled pyruvate or PEP with singly ^13^C-labeled CO_2_ and from the generation of singly ^13^C-labeled malate though the TCA cycle using malate dehydrogenase (Fig. 3C). The relative contributions of the two precursors to OAA (i.e., pyruvate/PEP versus malate) in P. protegens Pf-5 was resolved with the MFA, which determined a substantially higher fractional flux of pyruvate to OAA (70%) than the flux of malate to OAA (7.7%) (Fig. 4A). This low flux through malate dehydrogenase was accompanied by a high flux for the direct conversion of malate to pyruvate (63.4%), thus highlighting a very active pyruvate shunt in P. protegens Pf-5 (Fig. 4A). Moreover, the ^13^C-labeling patterns of TCA cycle metabolites implied an inactive glyoxylate shunt, which bypasses the decarboxylation reactions in the canonical TCA cycle to produce malate and succinate from citrate (see Fig. S5). Specifically, there was a lack of triply ^13^C-labeled succinate, which would be produced from triply and quadruply ^13^C-labeled citrate through the glyoxylate shunt (Fig. S5).

**(iv) Energetics of glucose catabolism.** To capture possible species-dependent differences in energetics generated directly from the central carbon metabolism, we compared the yields of reduced ubiquinone (UQH_2_), NAD(P)H, and ATP using our MFA-based cellular fluxes in P. protegens Pf-5 and those previously reported for P. putida KT2440, a well-studied biocatalyst candidate (10) (Fig. 4B). Compared to P. putida KT2440 (10), there was a slightly higher flux (approximately 5% higher) from glucose to gluconate in P. protegens Pf-5, but the flux from malate to OAA was lower (by approximately 25%) in P. protegens Pf-5. Accordingly, there was a higher yield of UQH_2_ from initial glucose catabolism in P. protegens Pf-5 but a higher UQH_2_ yield from the TCA cycle in P. putida KT2440 (Fig. 4B). For the yield of NAD(P)H, there was a higher contribution from the oxidative PP pathway (by approximately 60%) in P. putida KT2440 than in P. protegens Pf-5, but the contribution of the flux from malate to pyruvate via the malic enzyme was lower (by approximately 81%) in P. putida KT2440 than in P. protegens Pf-5; the contribution of the TCA cycle to NAD(P)H generation remained about the same in both species (Fig. 4B). With respect to ATP production by substrate-level phosphorylation, P. protegens Pf-5 produced less [by approximately 3 mmol ATP per cell dry weight in grams (g_CDW_)] than P. putida KT2440 because of the relatively lower fluxes in the downstream ED pathway of P. protegens (10) (Fig. 4). However, the net ATP yields were similar, because P. protegens Pf-5 consumed less ATP than P. putida KT2440 in the initial glucose catabolism due to the higher flux of glucose oxidation to gluconate and 2-ketogluconate and the subsequent carbon loss due to the secretions of these oxidized products in P. protegens Pf-5 (10) (Fig. 4). Remarkably, despite the different contributions of the relevant metabolic pathways, the combination of these contributions led to near-equivalent net yields of UQH_2_, NAD(P)H, and ATP in P. protegens Pf-5 and P. putida KT2440 (Fig. 4B).

### Hierarchy of glucose metabolism in the presence of other carbohydrates.

**FIG 5:**
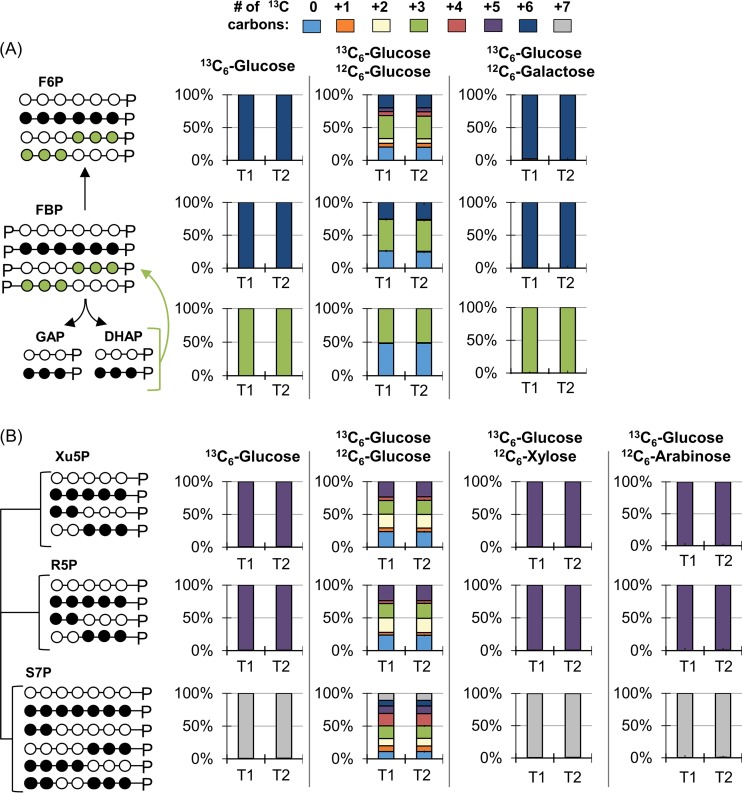
Metabolite labeling patterns during growth on [U-^13^C_6_]glucose (^13^C_6_-Glucose) alone or with unlabeled glucose (^12^C_6_-Glucose), unlabeled galactose (^12^C_6_-Galactose), unlabeled xylose (^12^C_6_-Xylose), or unlabeled arabinose (^12^C_6_-Arabinose). (A) Carbon mapping (left) and the labeling data (right) for intracellular metabolites in the upper Embden-Meyerhof-Parnas pathway following feeding on [^13^C_6_]glucose alone or with [^12^C_6_]glucose or [^2^C_6_]galactose. (B) Carbon mapping (left) and the labeling data (right) for intracellular metabolites in the pentose-phosphate pathway following feeding on [^13^C_6_]glucose alone or with [^12^C_6_]glucose, [^12^C_6_]xylose, or [^12^C_6_]arabinose. In the carbon mapping in panels A and B, the open circles and the filled circles represent nonlabeled and ^13^C-labeled carbons, respectively. Data were obtained at two time points during exponential growth: at OD_600_ 0.5 to 0.6 (T1) and at OD_600_ 0.9 to 1.0 (T2). Metabolite labeling data (average ± standard deviation) were from independent biological replicates (*n =* 3). Very small error bars are not noticeable. The metabolite abbreviations are as listed in legends for Fig. 1 and 3.

**FIG 6:**
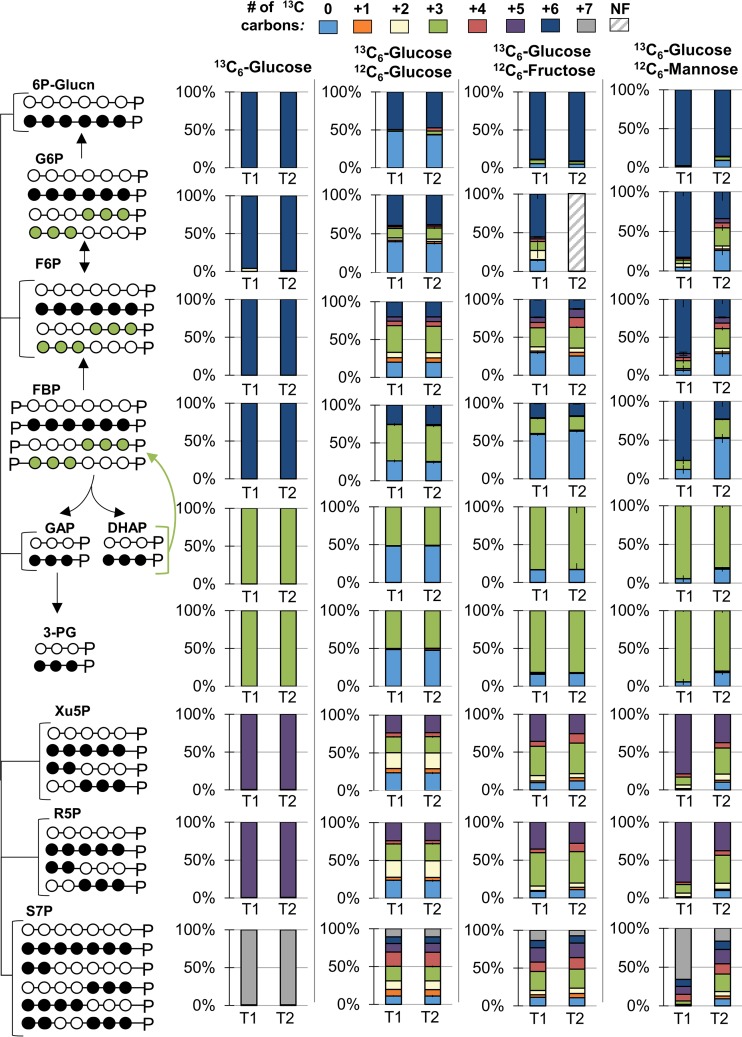
Metabolite labeling patterns during growth on [U-^13^C_6_]glucose (^13^C_6_-Glucose) alone or with unlabeled glucose (^12^C_6_-Glucose), unlabeled fructose (^12^C_6_-Fructose), or unlabeled mannose (^12^C_6_-Mannose). Carbon mapping (left) and the labeling data (right) for intracellular metabolites in the upper Embden-Meyerhof-Parnas (EMP) pathway, the pentose-phosphate pathway, and the Entner-Doudoroff pathway. The open circles and the filled circles represent nonlabeled and ^13^C-labeled carbons, respectively; the green circles represent labeling schemes specifically from reverse flux through upper EMP pathway. Data were obtained at two time points during exponential growth: at OD_600_ of 0.5 to 0.6 (T1) and at OD_600_ of 0.9 to 1.0 (T2). NF, not found. Labeling data (average ± standard deviation) were from independent biological replicates (*n =* 3). Very small error bars are not noticeable. The abbreviations are as listed in the legends for Fig. 1 and 3.

**(i) Proof of concept with ^13^C-labeled glucose and unlabeled glucose.** Before determining the relative incorporation of [U-^13^C_6_]glucose in the presence of a nonlabeled carbohydrate (xylose, arabinose, galactose, fructose, or mannose), we first conducted proof-of-concept experiments with the cells grown on [U-^13^C_6_]glucose alone or in a 1:1 mixture with unlabeled glucose (Fig. 5 and 6) (24). By a comparative analysis with the metabolite labeling patterns in the latter two conditions, we sought to determine the relative incorporation of other unlabeled carbohydrates in the presence of [U-^13^C_6_]glucose (Fig. 5 and 6).

**(ii) Glucose with xylose, arabinose, or galactose.** During growth on ^13^C-labeled glucose and galactose, the labeling patterns of metabolites in the EMP and ED pathways (F6P, FBP, and DHAP) were identical to the metabolite labeling during feeding on glucose alone, thus indicating the lack of galactose catabolism in the presence of glucose (Fig. 5A). During simultaneous feeding on ^13^C-labeled glucose and a pentose substrate (xylose or arabinose), the labeling patterns of metabolites in the PP pathway (Xu5P, R5P, and S7P) also indicated the lack of pentose assimilation (Fig. 5B). Furthermore, we found no incorporation of unlabeled xylose into α-ketoglutarate, consistent with the lack of the Weimberg pathway (see Fig. S6). The previously reported absence of P. protegens Pf-5 growth on medium containing only galactose, xylose, or arabinose implied the absence of assimilation of these carbohydrates (6). However, a recent study (21) showed extracellular xylose depletion by P. protegens Pf-5 during growth on a mixture of carbohydrates, suggesting that a lack of growth is not predictive of extracellular substrate depletion or possible assimilation. Our ^13^C-labeling data explicitly ascertained the absence of carbon assimilation from galactose, xylose, or arabinose in the presence of glucose (Fig. 5).

**(iii) Glucose with fructose.** Following growth simultaneously on [U-^13^C_6_]glucose and unlabeled fructose, there was a persistent presence of nonlabeled fractions in the intracellular metabolites, indicating both uptake and assimilation of fructose in the presence of glucose (Fig. 6). However, the high abundance of the nonlabeled fraction and the increased pool size of FBP compared to that in cells grown only on glucose indicated a bottleneck in fructose assimilation, which may explain the lower rate of fructose uptake (2.02 ± 0.86 mmol g_CDW_^−1^ · h^−1^) than glucose uptake (7.01 ± 1.70 mmol g_CDW_^−1^ · h^−1^) (Fig. 2B and 6; see also Fig. S7). In accordance with fructose incorporation through F1P into FBP, the highest fraction of nonlabeled carbons was in FBP (59% to 62%); both F6P and DHAP had lower nonlabeled fractions (25% to 30% and 17%, respectively) (Fig. 6). The lower fraction of nonlabeled carbons in DHAP than in F6P implied that the fructose-derived carbons were preferentially routed via a backward flux through upper EMP pathway (i.e., from FBP to G6P) toward the ED pathway (Fig. 6). The labeling of G6P reflected the nonlabeled and partially ^13^C-labeled fractions from F6P, consistent with this backward flux (Fig. 6). Therefore, in lieu of the forward EMP pathway (i.e., from G6P to FBP), our data stressed the importance of the ED pathway in the co-processing of fructose with glucose, and this was further verified below with ^13^C-MFA.

The labeling patterns of metabolites in the PP pathway also showed that the contribution of the fructose-derived carbons in this pathway was preferentially through the non-oxidative route, as previously shown in cells grown on glucose alone (Fig. 3 and 6). A transketolase reaction in the non-oxidative PP pathway combines the first two carbons of F6P with GAP to produce Xu5P. Both Xu5P and R5P have significant fractions of triply ^13^C-labeled carbons (38% to 44%), in accordance with the combination of nonlabeled F6P with triply ^13^C-labeled GAP following growth on ^13^C-labeled glucose with unlabeled fructose (Fig. 6). Due to the low fraction of nonlabeled GAP (as determined from DHAP labeling), there was a lack of doubly ^13^C-labeled R5P and Xu5P, which was also evident in cells grown on [U-^13^C_6_]glucose with unlabeled glucose (Fig. 6).

**(iv) Glucose with mannose.** In contrast to the metabolite labeling during growth on [U-^13^C_6_]glucose with unlabeled fructose, the metabolite labeling patterns following feeding on the [U-^13^C_6_]glucose with unlabeled mannose showed delayed assimilation of mannose into intracellular metabolism during exponential cell growth (Fig. 6). This was evident by the substantial increase in the nonlabeled fraction of FBP across the two time points, from 12% at an optical density at 600 nm (OD_600_) of ∼0.5 to 52% at OD_600_ of ∼1.0 (Fig. 6). This time-dependent labeling data matched with the initiation of a significant decrease in extracellular mannose concentration after 6 h of growth following the depletion of glucose (Fig. 2B).

A higher nonlabeled fraction of FBP than of F6P implied that the incorporation of mannose-derived carbons into FBP by way of fructose was preferred in P. protegens Pf-5 (Fig. 6). In agreement with this catabolic route for mannose, there was a larger pool of FBP than F6P when cells were grown on a mixture of glucose and mannose relative to when cells were grown on glucose only—this relatively larger FBP pool was also obtained in cells grown on a mixture of glucose and fructose (Fig. S7). Following growth on [^13^C]glucose with unlabeled mannose, the nonlabeled fraction of DHAP (18%) was lower than the corresponding fraction in F6P (28%) and G6P (26%) (Fig. 6). These labeling data indicated collectively a cycling of nonlabeled mannose carbons backward through the upper EMP pathway toward the ED pathway, similar to the intracellular metabolic route of fructose (Fig. 6).

### Cellular carbon fluxes during co-utilization of glucose and fructose.

**FIG 7:**
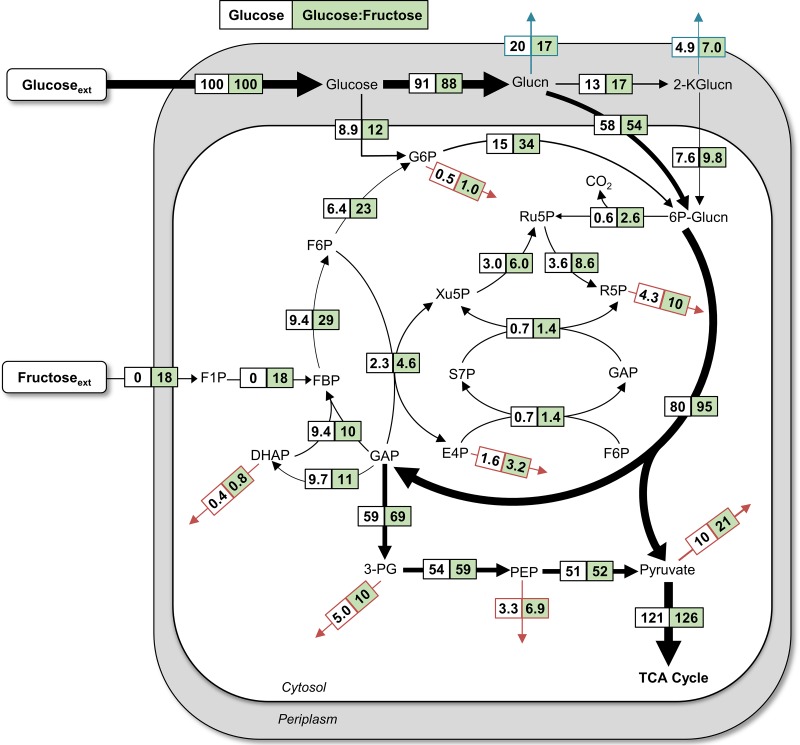
Quantitative metabolic flux analysis of P. protegens Pf-5 using metabolite labeling data following growth on [U-^13^C_6_]glucose and unlabeled glucose (white) or [U-^13^C_6_]glucose and unlabeled fructose (green). All fluxes were normalized to 100% glucose uptake, and the thickness of each arrow was scaled to the relative flux percentage for the glucose-only growth condition. Red arrows indicate contribution to biomass and blue arrows indicate excretion fluxes. The metabolite abbreviations are as listed in the legends for Fig. 1 and 3. The absolute fluxes (mean ± standard deviation), which were obtained by modeling experimental labeling data from three biological replicates, are listed in Tables S5 and S7.

Quantitative MFA was used to assess the differences in the metabolic fluxes during growth on glucose alone versus growth on glucose with fructose (Fig. 7; see also Tables S4 and S5). Our MFA focused on the pathways surrounding the cellular entry points of carbohydrates: initial glucose catabolism, the EMP pathway, the PP pathway, and the ED pathway (Fig. 7). Each MFA made use of the labeling data collected at an OD_600_ of 0.5 during early exponential phase before extracellular glucose is depleted and was constrained by the consumption rate of each carbohydrate, biomass effluxes, and metabolite secretions (Fig. 7 and S2; Tables S5 and S6). Upon optimization of the estimated metabolic fluxes, the model-estimated ^13^C-labeling patterns agreed well with the experimentally determined ^13^C-labeling patterns for each condition (see Fig. S8 and S9).

Despite the genome-encoded capabilities in P. protegens Pf-5 to split FBP into DHAP and GAP (7), the MFA revealed that the net flux was instead the aldolase reaction that combines DHAP and GAP to generate FBP (Fig. 7). Due to the additional incorporation of fructose-derived carbons through the flux from FBP to 6P-gluconate, there were higher fluxes from FBP to F6P (3-fold increase), F6P to G6P (3.6-fold increase), and G6P to 6P-gluconate (2.3-fold increase) in cells grown on the mixture of glucose and fructose compared to those grown on glucose alone (Fig. 7). Similar to the metabolism of glucose alone, there was a significant flux of the glucose uptake channeled through glucose oxidation to gluconate (88%) and the ED pathway (95%) during the metabolism of both glucose and fructose (Fig. 7). Consistent with the increased carbon flux toward 6P-gluconate from fructose assimilation, there was a 4.3-fold increase in the flux toward the oxidative PP pathway (i.e., from 6P-gluconate to Ru5P) and a 19% increase in the ED pathway (i.e., from 6-gluconate to GAP and pyruvate) (Fig. 7).

### Kinetic isotope analysis during co-utilization of glucose and mannose.

**FIG 8:**
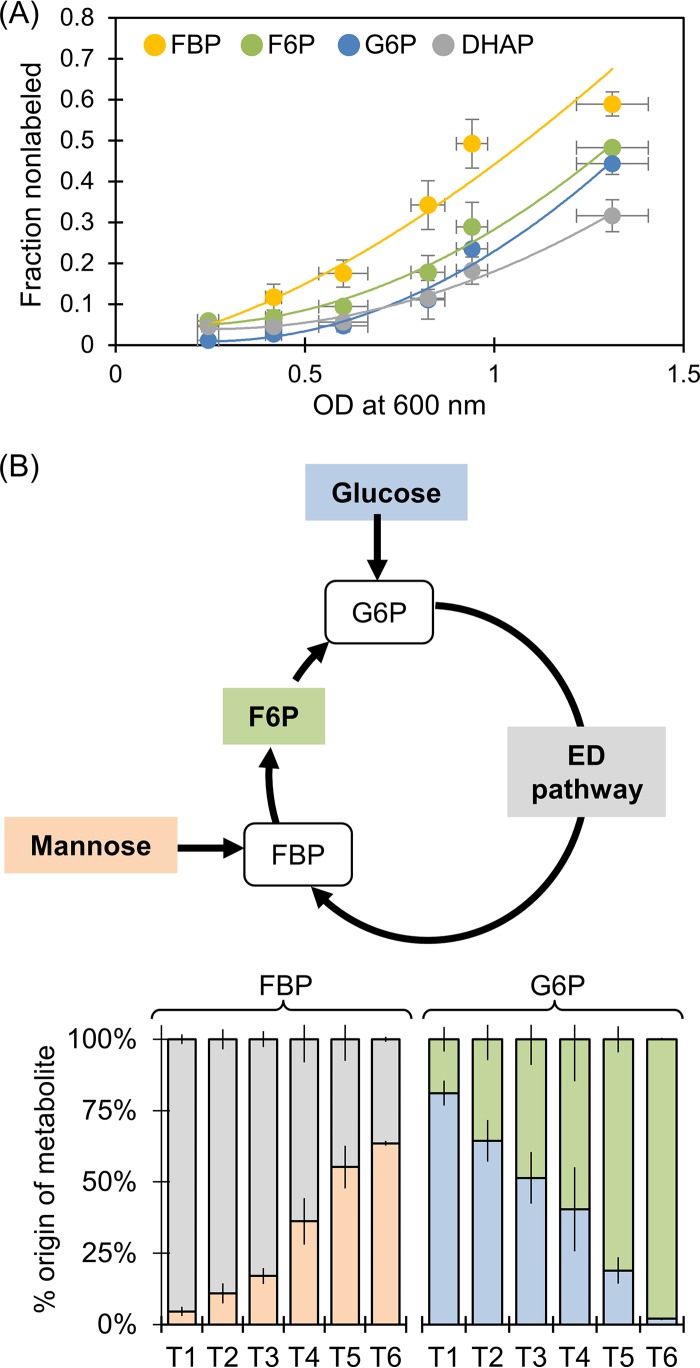
Metabolic routing of mannose through central metabolism in the presence of glucose. (A) Kinetic profiling of metabolite labeling patterns in cells grown simultaneously on [U-^13^C_6_]glucose and unlabeled mannose; the lines through the data are to guide the eye. (B) Dynamic flux ratio analysis for the metabolic entry point of glucose versus mannose assimilation through G6P and FBP, respectively: flux from mannose (light orange), flux from ED pathway (gray), flux from F6P (green), flux from glucose (blue). Data in panel B were obtained at six time points during exponential growth: at OD_600_ of 0.21 to 0.27 (T1), at OD_600_ of 0.40 to.44 (T2), at OD_600_ of 0.54 to 0.67 (T3), at OD_600_ of 0.79 to.87 (T4), at OD_600_ of 0.90 to 0.98 (T5), and at OD_600_ of 1.2 to 1.4 (T6). In panels A and B, The data (average ± standard deviation) are from biological replicates (*n =* 3). The metabolite abbreviations are as listed in the legend for Fig. 1.

Due to the lack of isotopic pseudo-steady state in metabolite labeling during growth on the glucose-mannose mixture, ^13^C-MFA could not be performed. To capture the assimilation route for mannose, we obtained labeling measurements at six time points during exponential growth on ^13^C-labeled glucose and unlabeled mannose (Fig. 8A; see also Fig. S10). Specifically, we examined the labeling patterns of gluconate, 6P-gluconate, G6P, F6P, FBP, and DHAP (Fig. 8A and S10). Across all time points, gluconate labeling was consistently ∼100% fully ^13^C labeled, indicating that gluconate was made exclusively from the ^13^C-labeled glucose (Fig. S10). However, the other five metabolites had nonlabeled fractions derived from the assimilation of unlabeled mannose (Fig. S10). Amongst all the metabolites, 6P-gluconate exhibited the slowest kinetic incorporation of nonlabeled fractions (Fig. S10). The appearance of a nonlabeled fraction in 6P-gluconate (starting at ∼4%) occurred at an OD_600_ of 0.94, the fifth time point (Fig. S10). In contrast, FBP exhibited the fastest incorporation of a nonlabeled fraction starting at an OD_600_ of 0.2 (Fig. 8A and S10). Compared to the labeling kinetics of FBP, there was a delay in the incorporation of nonlabeled carbons in F6P and G6P, which started to occur at an OD_600_ of 0.6, and DHAP, which steadily increased after an OD_600_ of 0.8 (Fig. 8A and S10). To determine whether there was statistical significance regarding how mannose-derived nonlabeled carbons were incorporated into metabolism, we analyzed the rates at which nonlabeled carbons were incorporated into FBP, F6P, G6P, and DHAP with a mixed-effects model. This statistical analysis (*F*_3,57_ = 12.973, *P* < 0.0001) confirmed that the significant effect of OD_600_ on the incorporation of mannose-derived nonlabeled carbons was dependent on the metabolite (Fig. 8A). Collectively, these kinetics data demonstrated that, instead of being channeled directly from FBP to GAP and DHAP, mannose carbons were incorporated at FBP and cycled up through F6P and G6P toward the ED pathway to generate subsequently GAP and DHAP (Fig. 8). Thus, the catabolic route for mannose was similar to what was determined for fructose catabolism.

Using metabolic flux ratio analysis across the different time points, we quantified the relative incorporation of glucose versus mannose at relevant entry points (G6P and FBP, respectively) into the metabolic network (Fig. 8B). The metabolite G6P is a result of fluxes from both direct glucose phosphorylation and from F6P derived from the cyclic ED-EMP pathway flux; the metabolite FBP is synthesized either from mannose or from downstream ED pathway flux through the combination of GAP and DHAP (Fig. 8B). In agreement with the extracellular depletion profiles (Fig. 2B), the fractional flux of glucose directly to G6P started at 81% at an OD_600_ of 0.2 and steadily decreased over time to reach eventually 2% at an OD_600_ of 1.2 (Fig. 8B). Conversely, the relative contribution of mannose-derived FBP went from 5% to eventually 64% at the highest measured OD_600_ (Fig. 8B). The cycling of carbons from mannose toward the ED pathway maintained a 36% fractional flux of triose phosphates to FBP, even after glucose was depleted (Fig. 8). In sum, the metabolic flux ratio analysis demonstrated simultaneous assimilation of both glucose and mannose into the intracellular metabolites, but the relative contribution of each substrate varied as a function of time (Fig. 8B).

## DISCUSSION

The metabolic networks and fluxes of several *Pseudomonas* species, including P. putida, P. fluorescens, and P. aeruginosa, have been previously described (8–11, 22, 25). Here, we present the first metabolic flux analysis of the recently characterized P. protegens Pf-5, a common plant commensal bacterium known to secrete specialized metabolites important for the biocontrol of fungi and bacteria pathogenic to plants (4, 6). Metabolic flux quantitation determined that initial glucose catabolism in P. protegens Pf-5 was primarily through periplasmic oxidation to gluconate, with relatively minor influx of glucose through G6P (Fig. 4A). Up to 95% of consumed glucose in P. protegens Pf-5 was channeled through the ED pathway, which was also reported in P. putida, P. fluorescens, and P. aeruginosa (8, 11, 22, 25). Further glucose catabolism was routed through a cyclic flux from the ED pathway to the upper EMP pathway, which was also elucidated in P. putida KT2440 (10, 11). Furthermore, the non-oxidative route was more significant than the oxidative route in generating PP pathway intermediates in P. protegens Pf-5, as previously reported for P. putida KT2440 (11) (Fig. 3B and 4A). The highly active flux of pyruvate formation from malate in the P. protegens Pf-5 cells was also reported in P. putida and P. fluorescens (8, 10, 22). In sum, our results stressed that the metabolic network for glucose metabolism in P. protegens Pf-5 is consistent with the metabolic network of previously studied species of the *Pseudomonas* genus. Finally, through the contribution of different metabolic pathways, the total UQH_2_, NAD(P)H, and ATP yields of P. protegens Pf-5 were remarkably similar to those of P. putida KT2440, whose metabolism has been featured for its capability of fulfilling high demands of reducing power (10).

Root exudates and the breakdown of polysaccharides from plant biomass both provide various carbohydrates that stimulate the growth of soil microorganisms, including *Pseudomonas* species (26). With respect to the catabolism of other carbohydrates besides glucose, we found that P. protegens Pf-5 did not metabolize the common hemicellulose monomers galactose, xylose, or arabinose in the presence of glucose but did utilize the carbon mixtures of glucose with fructose or mannose (Fig. 5 and 6). The lack of assimilation of the pentoses and galactose was consistent with the absence of genes for the relevant catabolic pathways. The preference for glucose over fructose and mannose implied catabolite repression by glucose. An elucidation of the underlying mechanism of this repression requires further transcriptional expression and mutation studies. In accordance with gene annotations, metabolite labeling data confirmed that fructose was incorporated into metabolism via FBP (Fig. 6). However, contrary to the possible route for mannose assimilation through F6P annotated in the P. protegens Pf-5 genome (7), the primary route of mannose assimilation in P. protegens Pf-5 was found to be also via FBP (Fig. 6). In addition, the appearance of fructose extracellularly during growth on the mixture of glucose and mannose implied a conversion of mannose to fructose prior to intracellular metabolism (Fig. 2B). Mannose conversion to fructose by a mannose isomerase has been reported previously in P. cepacia, P. aeruginosa, and P. saccharophila (17–20). Whether a nonspecific isomerase exists in P. protegens Pf-5 remains to be determined.

The cyclic metabolism linking backward flux from FBP through the upper EMP pathway toward the ED pathway produces equivalent amounts of NADPH, but less net ATP, compared to that with direct FBP conversion to GAP and DHAP. Interestingly, instead of this direct contribution to the lower EMP pathway, carbons from assimilated fructose and mannose were routed through the cyclic connection between the upper EMP pathway and the ED pathway during mixed-hexose utilization (Fig. 7 and 8). Comparable MFA findings were reported for fructose-only catabolism in P. putida KT2440 and P. fluorescens SBW25 (14, 16). Therefore, P. protegens Pf-5 exhibits a strong reliance on the ED pathway for both fructose and mannose assimilation even in the presence of glucose.

Similar growth phenotypes during co-utilization of hexoses implied that, despite different carbohydrates in the growth medium at the same total carbon content, P. protegens Pf-5 preserved a constant biomass (Fig. 2A). However, the uptake of glucose was preferred over the uptake of fructose (3 to 1) or mannose (4 to 1) (Fig. 2; see also Table S2 in the supplemental material). The composition ratio of glucose to fructose in maize root exudates was found to be 2 to 1 (27). Across soil horizons, the glucose-mannose ratio ranged approximately from 3:1 to 5:1 (28). Therefore, our relative consumption rates of glucose versus fructose or mannose in P. protegens Pf-5 were remarkably in agreement with the relative composition of these carbohydrates in environmentally relevant conditions. Whether this agreement is due to an evolutionary connection to nutritional adaptability warrants further investigation.

In regards to the potential of P. protegens Pf-5 as a biocatalytic platform, the innate production and secretion of gluconate and 2-ketogluconate in P. protegens Pf-5 are attractive features. Oxidized sugars are important precursors to polymeric materials, including polyesters. In fact, gluconate was identified as a top 30 value-added candidate for the production of bio-inspired materials (29). Under our experimental conditions, the quantitative flux analysis determined that the secretion rates of gluconate and 2-ketogluconate collectively accounted for approximately 35% of the glucose uptake in P. protegens during exponential growth, whereas metabolite secretion of gluconate was reported to be less than 10% of the glucose uptake in P. putida KT2440 (11) (Fig. 2C). While there were relatively similar secretions during growth on glucose alone or on glucose and fructose, growth on glucose and mannose resulted in a decrease in the total secretion (by approximately 3.5 mM) of gluconate and 2-ketogluconate (Fig. 2C). After glucose was depleted, decreases in the concentrations of both gluconate and 2-ketogluconate indicated that the cells can utilize these metabolites once their favored carbon source (i.e., glucose) was absent (Fig. 2B and C). This phenomenon would need to be considered and manipulated to harvest these metabolite secretions in P. protegens Pf-5 as valuable products. In light of the abundance of different types of carbohydrates in natural environments and in renewable carbon feedstocks used for engineered bioproduction (30), our findings collectively provide important insights regarding the cellular metabolism underlying carbohydrate co-utilization in P. protegens Pf-5 and related *Pseudomonas* species.

## MATERIALS AND METHODS

### Materials.

The P. protegens Pf-5 cells were acquired from the American Type Culture Collection (Manassas, VA). Unless noted otherwise, the chemicals used in the growth media were obtained from Sigma-Aldrich (St. Louis, MO), Cayman Chemical (Ann Arbor, MI), or Fisher Scientific (Pittsburgh, PA). The ^13^C-labeled glucose ([U-^13^C_6_]glucose and [1,2-^13^C_6_]glucose) were purchased from Cambridge Isotopes (Tewksbury, MA) and Omicron Biochemicals (South Bend, IN), respectively. All culture solutions were prepared with Millipore water (18.2 MΩ·cm; Millipore, Billerica, MA, USA) while resuspensions for LC-HRMS analysis were made with LC-MS-grade water. Solutions were sterilized by passing through a 0.22-μm nylon filters (Waters Corporation, MA). An Agilent Cary UV-visible spectrophotometer (Santa Clara, CA) was used for optical density readings at 600 nm. The LC-HRMS analysis was conducted on an ultra-high-performance LC (Dionex UltiMate 3000; Thermo Scientific) coupled to a high-resolution accurate-mass MS (Q Exactive quadrupole-Orbitrap hybrid MS; Thermo Scientific) with electrospray ionization.

### Culturing conditions and growth measurements.

Batch growth experiments (three to seven biological replicates) of P. protegens Pf-5 were conducted in an incubator (model I24; New Brunswick Scientific, Edison, NJ) maintained at 30°C and shaken at 220 rpm. The initial growth in nutrient-rich medium prior to growth in minimal-nutrient medium was conducted as previously described (11). Final growth experiments were conducted in 125-ml baffled flasks with a pH-adjusted (7.0) filter-sterilized minimal-nutrient medium that contained major salts and essential trace metal nutrients as previously reported (31): 89.4 mM K_2_HPO_4_, 56.4 mM NaH_2_PO_4_, 0.81 mM MgSO_4_·7H_2_O, 18.7 mM NH_4_Cl, 8.6 mM NaCl, 34 μM CaCl_2_·2H_2_O, 30 μM FeSO_4_·7H_2_O, 0.86 μM CuSO_4_·5H_2_O, 1.9 μM H_3_BO_3_, 7.7 μM ZnSO_4_·7H_2_O, 0.75 μM MnSO_4_·5H_2_O, 0.26 μM NiCl_2_·6H_2_O, and 0.3 1 μM Na_2_MoO_4_·5H_2_O. The carbohydrate composition was 100 mM C total for glucose alone (equivalent to 16.7 mM or 3 g·liter^−1^ glucose) and for 1:1 mixtures of glucose and xylose, glucose and arabinose, glucose and galactose, glucose and mannose, or glucose and fructose. For cellular isotopic enrichment, either [U-^13^C_6_]glucose or [1,2-^13^C_6_]glucose was used in glucose-only growth, but only [U-^13^C_6_]glucose was used for mixtures in combination with an unlabeled second carbohydrate. Bacterial growth in the biological replicates was monitored as a function of time until late stationary phase using OD_600_ measurements (see Fig. S1 in the supplemental material)—cell suspensions were diluted when the OD_600_ value was above 0.5 to get accurate readings. Cell dry weight in grams was also determined throughout growth by lyophilizing the cell pellets as previously described (11). Biomass growth rate (per hour) for each growth condition was obtained by regression analysis of OD_600_ measurements over time (Table S2).

### Measurement of carbohydrate consumption.

Independent ^13^C-tracer experiments, as described in “Metabolite monitoring and quantification,” confirmed that extracellular depletion of the substrates correlated with substrate consumption. The extracellular depletion of each carbohydrate substrate (three biological replicates) was determined throughout 24 h of cell growth. Culture aliquots were pelleted by centrifugation, and the supernatants were stored at −20°C until further analysis. According to previously reported LC methods for carbohydrate analysis (21), we applied an analytical method using LC-HRMS for monitoring the carbohydrate concentration in the extracellular solution. Peak identification and quantification of carbohydrate concentrations were conducted with Thermo Scientific Xcalibur 3.0 Quan Browser. Carbohydrate consumption rates (in millimoles per gram [dry weight] per hour) were obtained by combining the regression analyses of carbohydrate depletion over time with biomass growth rates (Table S2).

### Metabolite monitoring and quantification.

**(i) Extracellular metabolites.** To determine metabolite excretion rates, cell suspension samples (three biological replicates) were harvested periodically throughout growth and pelleted with centrifugation, and the supernatants were removed and stored at −20°C until LC-HRMS analysis. Dilutions of 1:10, 1:100, and 1:1,000 were conducted to account for the various concentrations of each metabolite over time. For the LC, an Acquity UPLC Waters 1.7-µm particle size column with dimensions of 2.1 mm by 100 mm was used for all metabolomics samples (Milford, MA), with a constant column temperature of 25°C. The flow rate was kept constant at 0.180 ml·min^−1^. The mobile phase composition and LC protocol were as previously described (31). The injection volume for each sample was 10 µL. The MS was operated in full-scan negative mode. Metabolite identification was based on accurate mass and matches with standard retention times. Metabolite levels were quantified using Thermo Scientific Xcalibur 3.0 Quan Browser.

**(ii) Intracellular metabolites.** Cells were separated by filtration and then lysed to extract the intracellular metabolites as described in Sasnow et al. (11). Metabolites in solution were monitored by LC-HRMS, and the ^13^C labeling patterns were analyzed on Metabolomic Analysis and Visualization Engine (MAVEN) software (32, 33). Isotopologue data were obtained for the following compounds: 6P-gluconate, G6P, F6P, FBP, DHAP, 3-phosphoglycerate, PEP, pyruvate, Xu5P, R5P, S7P, aspartate, citrate, α-ketoglutarate, succinate, and malate. Aspartate ^13^C labeling was used as a proxy for OAA ^13^C labeling by assuming equilibrium between the two compounds (11). The labeling of dissolved CO_2_ was estimated from the labeling patterns of ornithine and citrulline (Fig. S2); ornithine incorporates one mole of dissolved CO_2_ to become citrulline. All the extracted isotopologues were corrected for natural abundance of ^13^C. To verify pseudo-steady-state isotopic enrichment of the intracellular pools, metabolites were isolated from cellular extracts obtained at two different time points during the exponential phase, at OD_600_ values of ∼0.5 and ∼1.0 (11). To analyze mannose incorporation over time, cells were extracted at six time points during exponential growth corresponding to OD_600_ values of ∼0.2, ∼0.4, ∼0.6, ∼0.8, ∼0.9, and ∼1.3. A mixed-effects model was conducted using R (34) and the lmerTest package (35), which modeled the nonlabeled fraction (log transformed) by OD_600_, metabolite, and their interaction with the random effect of biological replicates.

### Quantitative metabolic flux modeling.

The quantitation of the metabolic fluxes was achieved for cells grown on [1,2-^13^C_2_]glucose alone, [U-^13^C_6_]glucose and unlabeled glucose, or [U-^13^C_6_]glucose and unlabeled fructose. Additionally, we employed the following experimental data to constrain the metabolic flux analysis: substrate uptake rates, metabolite excretions, growth rate, and cellular stoichiometry. To determine the uptake of glucose-derived carbons from the periplasm to the cytosol (to add as constraints in the flux modeling), a regression analysis was conducted on the extracellular glucose depletion after subtracting the extracellular amounts of both gluconate and 2-ketogluconate (Table S2; Fig. 2). We constrained the model to account for periplasmic oxidation reactions and secretion rates based on the adjusted substrate uptake rates into cytosol (Fig. S2). Carbon effluxes from intermediates in central metabolism toward biomass production were determined on the basis of the growth rate under each condition and the biomass composition of P. putida (nucleic acids, proteins, cell membrane, and carbohydrate polymers) (36). An initial reaction network for the central carbon metabolism of P. protegens PF-5 was constructed using a predicted genome-scale metabolic model (7) and gene annotation of metabolic enzymes reported on the KEGG database (37–39) and MetaCyc (40). The metabolic reaction network was validated through ^13^C labeling of intracellular metabolites. The following reactions were constrained in the forward direction: gluconate → 6P-gluconate, 6P-gluconate → ribulose 5-phosphate, gluconate → 2-ketogluconate, 2-ketogluconate → 6P-gluconate, glucose → G6P, FBP → F6P, malate → pyruvate, pyruvate → OAA, and PEP → OAA. Optimized fluxes in the model metabolic network reactions were determined by the 13CFLUX2 software package (http://www.13cflux.net) (41), whereby the quality of fit was optimized iteratively by comparing experimental ^13^C-labeling data and the *in silico*-estimated labeling data.

For cells grown on [U-^13^C_6_]glucose and mannose, we conducted a metabolic flux ratio analysis across six time points during exponential growth (see Fig. S11). Specifically, we determined the relative flux contributions of substrate uptake versus intracellular metabolic reactions to the biosynthesis of G6P and FBP (Fig. S11). Optimized flux ratios were obtained by iterative optimization with the objective of obtaining the minimum error difference between experimental and simulated labeling data (Fig. S11).

## Supplementary Material

Supplemental file 1

## References

[1] Singh PB, Saini HS, Kahlon RS 2016 *Pseudomonas*: the versatile and adaptive metabolic network, p 81–126. *In* Kahlon RS (ed), Pseudomonas: molecular and applied biology. Springer International Publishing, Cham, Switzerland.

[2] Hung C-S, Zingarelli S, Nadeau LJ, Biffinger JC, Drake CA, Crouch AL, Barlow DE, Russell JN, Crookes-Goodson WJ 2016 Carbon catabolite repression and Impranil polyurethane degradation in *Pseudomonas protegens* strain Pf-5. Appl Environ Microbiol 82:6080–6090. doi:10.1128/AEM.01448-16. 27496773 PMC5068165

[3] Loper JE, Gross H 2007 Genomic analysis of antifungal metabolite production by *Pseudomonas fluorescens* Pf-5. Eur J Plant Pathol 119:265–278. doi:10.1007/s10658-007-9179-8.

[4] Loper JE, Kobayashi DY, Paulsen IT 2007 The genomic sequence of *Pseudomonas fluorescens* Pf-5: insights into biological control. Phytopathology 97:233–238. doi:10.1094/PHYTO-97-2-0233. 18944380

[5] Nowak-Thompson B, Gould J, Kraus J, Loper E 1994 Production of 2,4-diacetylphloroglucinol by the biocontrol agent *Pseudomonas fluorescens* Pf-5. Can J Microbiol 40:1064–1066. doi:10.1139/m94-168.

[6] Ramette A, Frapolli M, Saux MF, Gruffaz C, Meyer J, Défago G, Sutra L, Moënne-Loccoz Y 2011 *Pseudomonas protegens* sp. nov., widespread plant-protecting bacteria producing the biocontrol compounds 2,4-diacetylphloroglucinol and pyoluteorin. Syst Appl Microbiol 34:180–188. doi:10.1016/j.syapm.2010.10.005. 21392918

[7] Paulsen IT, Press CM, Ravel J, Kobayashi DY, Myers GSA, Mavrodi DV, DeBoy RT, Seshadri R, Ren Q, Madupu R, Dodson RJ, Durkin AS, Brinkac LM, Daugherty SC, Sullivan SA, Rosovitz MJ, Gwinn ML, Zhou L, Schneider DJ, Cartinhour SW, Nelson WC, Weidman J, Watkins K, Tran K, Khouri H, Pierson EA, Pierson LS, Thomashow LS, Loper JE 2005 Complete genome sequence of the plant commensal *Pseudomonas fluorescens* Pf-5. Nat Biotechnol 23:873–878. doi:10.1038/nbt1110. 15980861 PMC7416659

[8] Del Castillo T, Ramos JL, Rodríguez-Herva JJ, Fuhrer T, Sauer U, Duque E 2007 Convergent peripheral pathways catalyze initial glucose catabolism in *Pseudomonas putida*: genomic and flux analysis. J Bacteriol 189:5142–5152. doi:10.1128/JB.00203-07. 17483213 PMC1951859

[9] Chavarría M, Kleijn RJ, Sauer U, Pflüger-Grau K, Lorenzo VD 2012 Regulatory tasks of the phosphoenolpyruvate-phosphotransferase system of *Pseudomonas putida* in central carbon metabolism. mBio 3:e00028-12. doi:10.1128/mBio.00028-12. 22434849 PMC3312210

[10] Nikel PI, Chavarría M, Fuhrer T, Sauer U, Lorenzo VD 2015 *Pseudomonas putida* KT2440 metabolizes glucose through a cycle formed by enzymes of the Entner-Doudoroff, Embden-Meyerhof-Parnas, and pentose phosphate pathways. J Biol Chem 290:25920–25932. doi:10.1074/jbc.M115.687749. 26350459 PMC4646247

[11] Sasnow SS, Wei H, Aristilde L 2016 Bypasses in intracellular glucose metabolism in iron-limited *Pseudomonas putida*. Microbiologyopen 5:3–20. doi:10.1002/mbo3.287. 26377487 PMC4767421

[12] Van Dijken JP, Quayle JR 1977 Fructose metabolism in four *Pseudomonas* species. Arch Microbiol 114:281–286. doi:10.1007/BF00446874. 143919

[13] Rojo F 2010 Carbon catabolite repression in *Pseudomonas*: optimizing metabolic versatility and interactions with the environment. FEMS Microbiol Rev 34:658–684. doi:10.1111/j.1574-6976.2010.00218.x. 20412307

[14] Lien SK, Niedenführ S, Sletta H, Nöh K, Bruheim P 2015 Fluxome study of *Pseudomonas fluorescens* reveals major reorganisation of carbon flux through central metabolic pathways in response to inactivation of the anti-sigma factor MucA. BMC Syst Biol 9:6. doi:10.1186/s12918-015-0148-0. 25889900 PMC4351692

[15] Sawyer MH, Baumann P, Baumann L, Berman SM, Canovas JL, Berman RH 1977 Pathways of d-fructose catabolism in species of *Pseudomonas*. Arch Microbiol 112:49–55. doi:10.1007/BF00446653. 139135

[16] Sudarsan S, Dethlefsen S, Blank LM, Siemann-Herzberg M, Schmid A 2014 The functional structure of central carbon metabolism in *Pseudomonas putida* KT2440. Appl Environ Microbiol 80:5292–5303. doi:10.1128/AEM.01643-14. 24951791 PMC4136102

[17] Eagon RG, Williams AK 1960 Enzymatic patterns of adaptation to fructose, glucose, and mannose exhibited by *Pseudomonas aeruginosa*. J Bacteriol 79:90–94. 13819088 10.1128/jb.79.1.90-94.1960 PMC278639

[18] Hu X, Shi Y, Zhang P, Miao M, Zhang T, Jiang B 2016 d-Mannose: properties, production, and applications: an overview. Compr Rev Food Sci Food Saf 15:773–785. doi:10.1111/1541-4337.12211. 33401842

[19] Allenza P, Morrell MJ, Detroy RW 1990 Conversion of mannose to fructose by immobilized mannose isomerase from *Pseudomonas cepacia*. Appl Biochem Biotechnol 24:171–182. doi:10.1007/BF02920243. 2353810

[20] Palleroni NJ, Doudoroff M 1956 Mannose isomerase of *Pseudomonas saccharophila*. J Biol Chem 218:535–548. 13278359

[21] Barzen-Hanson KA, Wilkes RA, Aristilde L 2018 Quantitation of carbohydrate monomers and dimers by liquid chromatography coupled with high-resolution mass spectrometry. Carbohydr Res 468:30–35. doi:10.1016/j.carres.2018.08.007. 30121416

[22] Fuhrer T, Fischer E, Sauer U 2005 Experimental identification and quantification of glucose metabolism in seven bacterial species. J Bacteriol 187:1581–1590. doi:10.1128/JB.187.5.1581-1590.2005. 15716428 PMC1064017

[23] Asai T, Aida K, Sugisaki Z, Yakeishi N 1955 On α-ketoglutaratic acid fermentation. J Gen Appl Microbiol 1:308–346. doi:10.2323/jgam.1.308.

[24] Aristilde L 2017 Metabolite labelling reveals hierarchies in *Clostridium acetobutylicum* that selectively channel carbons from sugar mixtures towards biofuel precursors. Microb Biotechnol 10:162–174. doi:10.1111/1751-7915.12459. 27878973 PMC5270725

[25] Berger A, Dohnt K, Tielen P, Jahn D, Becker J, Wittmann C 2014 Robustness and plasticity of metabolic pathway flux among uropathogenic isolates of *Pseudomonas aeruginosa*. PLoS One 9:e88368. doi:10.1371/journal.pone.0088368. 24709961 PMC3977821

[26] Gunina A, Kuzyakov Y 2015 Sugars in soil and sweets for microorganisms: review of origin, content, composition and fate. Soil Biol Biochem 90:87–100. doi:10.1016/j.soilbio.2015.07.021.

[27] Hütsch BW, Augustin J, Merbach W 2002 Plant rhizodeposition—an important source for carbon turnover in soils. J Plant Nutr Soil Sci (1999) 165:397. doi:10.1002/1522-2624(200208)165:4<397::AID-JPLN397>3.0.CO;2-C.

[28] Benzing-Purdie L 1980 Organic matter and carbohydrate distribution in an orthic humic gleysol. Soil Biol Biochem 12:567–571. doi:10.1016/0038-0717(80)90037-1.

[29] Werpy T, Petersen G, Aden A, Bozell J, Holladay J, White J, Manheim A, Eliot D, Lasure L, Jones S 2004 Top value-added chemicals from biomass. Volume 1–results of screening for potential candidates from sugars and synthesis gas. U.S. Department of Energy Efficiency and Renewable Energy, Washington, DC.

[30] Poblete-Castro I, Becker J, Dohnt K, dos Santos VM, Wittmann C 2012 Industrial biotechnology of *Pseudomonas putida* and related species. Appl Microbiol Biotechnol 93:2279–2290. doi:10.1007/s00253-012-3928-0. 22350258

[31] Aristilde L, Reed ML, Wilkes RA, Youngster T, Kukurugya MA, Katz V, Sasaki CRS 2017 Glyphosate-induced specific and widespread perturbations in the metabolome of soil *Pseudomonas* species. Front Environ Sci 5:34. doi:10.3389/fenvs.2017.00034.

[32] Clasquin MF, Melamud E, Rabinowitz JD 2012 LC-MS data processing with MAVEN: a metabolomic analysis and visualization engine. Curr Protoc Bioinformatics Chapter 14:Unit14.11. doi:10.1002/0471250953.bi1411s37. PMC4055029 22389014

[33] Melamud E, Vastag L, Rabinowitz JD 2010 Metabolomic analysis and visualization engine for LC−MS data. Anal Chem 82:9818–9826. doi:10.1021/ac1021166. 21049934 PMC5748896

[34] R Core Team. 2016 R: a language and environment for statistical computing. R Foundation for Statistical Computing, Vienna, Austria.

[35] Kuznetsova A, Brockhoff P, Christensen R 2017 lmerTest package: tests in linear mixed effects models. J Stat Softw 82:1–26.

[36] van Duuren JB, Puchałka J, Mars AE, Bücker R, Eggink G, Wittmann C, dos Santos VA 2013 Reconciling *in vivo* and *in silico* key biological parameters of *Pseudomonas putida* KT2440 during growth on glucose under carbon-limited condition. BMC Biotechnol 13:93. doi:10.1186/1472-6750-13-93. 24168623 PMC3829105

[37] Kanehisa M, Sato Y, Kawashima M, Furumichi M, Tanabe M 2016 KEGG as a reference resource for gene and protein annotation. Nucleic Acids Res 44:D457–D462. doi:10.1093/nar/gkv1070. 26476454 PMC4702792

[38] Kanehisa M, Goto S 2000 KEGG: Kyoto encyclopedia of genes and genomes. Nucleic Acids Res 28:27–30. doi:10.1093/nar/28.1.27. 10592173 PMC102409

[39] Kanehisa M, Furumichi M, Tanabe M, Sato Y, Morishima K 2017 KEGG: new perspectives on genomes, pathways, diseases and drugs. Nucleic Acids Res 45:D353–D361. doi:10.1093/nar/gkw1092. 27899662 PMC5210567

[40] Caspi R, Altman T, Dreher K, Fulcher CA, Subhraveti P, Keseler IM, Kothari A, Krummenacker M, Latendresse M, Mueller LA, Ong Q, Paley S, Pujar A, Shearer AG, Travers M, Weerasinghe D, Zhang P, Karp PD 2012 The MetaCyc database of metabolic pathways and enzymes and the BioCyc collection of pathway/genome databases. Nucleic Acids Res 40:D742–D753. doi:10.1093/nar/gkr1014. 22102576 PMC3245006

[41] Weitzel M, Nöh K, Dalman T, Niedenführ S, Stute B, Wiechert W 2013 13CFLUX2—high-performance software suite for (13)C-metabolic flux analysis. Bioinformatics 29:143–145. doi:10.1093/bioinformatics/bts646. 23110970 PMC3530911

