# Supplementary material for: A Cyclic Metabolic Network in Pseudomonas protegens Pf-5 Prioritizes the Entner-Doudoroff Pathway and Exhibits Substrate Hierarchy during Carbohydrate Co-Utilization
Source: Appl Environ Microbiol. 2018 Dec 13;85(1):e02084-18. doi: 10.1128/AEM.02084-18 (PMC6293094; doi:10.1128/AEM.02084-18)
Supplement: Supplemental file 1 [file AEM.02084-18-s0001.pdf]

Journal: *Applied and Environmental Microbiology*
Section: Physiology

A Cyclic Metabolic Network in *Pseudomonas protegens* Pf-5 Prioritizes the Entner-Doudoroff Pathway and Exhibits Substrate Hierarchy during Carbohydrate Co-Utilization

Authors: Rebecca A. Wilkes[a], Caroll M. Mendonca[a], and Ludmilla Aristilde[a,#]
Affiliation: [a]Department of Biological and Environmental Engineering, College of Agriculture and Life Sciences, Cornell University, Ithaca, NY 14853, USA

[#]Corresponding author:
214 Riley-Robb Hall, Cornell University, Ithaca, NY 14850
Phone: (607) 255-6845. Fax: (607) 255-4449. E-mail: ludmilla@cornell.edu

SUPPLEMENTARY INFORMATION

Table S1
Fig. S1
Table S2
Fig. S2
Table S3
Table S4
Fig. S3
Fig. S4
Fig. S5
Fig. S6
Fig. S7
Table S5
Table S6
Fig. S8
Fig. S9
Fig. S10
Fig. S11

Table S1. List of the corresponding gene loci and gene annotations to the reactions and genes shown in Fig. 1 in the main text. NF indicates gene name was not found..

Reactions	Gene	Gene locus	Annotation
$Gluc_{ext}$ -> $Gluc_{peri}$	*oprB*	PFL_3366	carbohydrate-selective porin
$Fruc_{ext}$ -> F1P	*FruA; FruB*	PFL_0861; PFL_0859	PTS system, fructose-specific IIBC component; putative multiphosphoryl transfer protein
F1P -> FBP	*FruK*	PFL_0860	1-phosphofructokinase
$Mann_{ext}$ -> M6P	*FruA; nagE; treP*	PFL_0861; PFL_1078; PFL_4934	PTS system, fructose-specific IIBC component; PTS system, N-acetylglucosamine-specific IIBC component; PTS system trehalose-specific IIBC component
M6P -> F6P	*pslB; algA*	PFL_4209; PFL_1013; PFL_5483	mannose-1-phosphate guanylyltransferase/mannose-6-phosphate isomerase; alginate biosynthesis protein; mannose-1-phosphate guanylyltransferase/mannose-6-phosphate isomerase
$Gluc_{peri}$ -> G6P	*glk*	PFL_4621	glucokinase
$Gluc_{peri}$ -> $Glucn_{peri}$	*gcd*	PFL_4916	quinoprotein glucose dehydrogenase
$Glucn_{peri}$-> 6P-Glucn	*gntT; gntK*	PFL_4579; PFL_4580	high-affinity gluconate transporter; gluconokinase
$Glucn_{peri}$ -> 2-$ketoglucn_{peri}$	NF	PFL_0053; PFL_0054; PFL_0055	gluconate 2-dehydrogenase subunit 3; gluconate 2-dehydrogenase flavoprotein; gluconate 2-dehydrogenase cytochrome c subunit
2-$ketoglucn_{peri}$ -> 2-$Ketoglucn_{cyt}$	NF	PFL_2718	MFS transporter, anion:cation symporter (ACS) fanily
2-ketoglucn -> 2-ketoglucnP	*kdgK_1*	PFL_2719	2-dehydro-3-deoxygluconokinase
2-$ketoglucn_{P}$ -> 6P-Glucn	*ghrB1*	PFL_2717	glyoxylate/hydroxypyruvate reductase B
G6P -> 6P-Glucn	*zwf; pgl*	PFL_3143; PFL_4609	glucose-6-phosphate dehydrogenase; 6-phosphogluconolactonase
DHAP -> GAP	*tpiA*	PFL_0839	triose-phosphate isomerase
FBP -> DHAP + GAP	*fba*	PFL_5781	fructose-bisphosphate aldolase, class II
FBP -> F6P	*fbp*	PFL_0393	fructose-1,6-bisphosphatase
F6P -> G6P	*pgi*	PFL_5280	glucose-6-phosphate isomerase
Xyl_{peri} -> Xyl_{cyt}	*rbsA; rbsB; rbsC; rbsD*	PFL_2102; PFL_2101; PFL_2103; PFL_2106	ribose ABC transporter, ATP-binding protein; ribose ABC transporter, periplasmic; ribose ABC transporter, permease protein; D-ribose pyranase

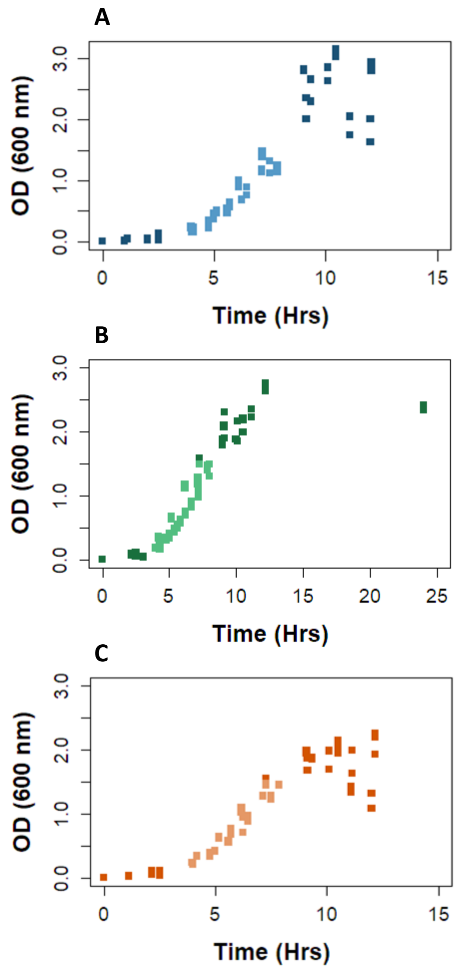

Fig. S1. Growth curves when grown on 100 mM total carbon of A) glucose alone (blue), B) glucose in 50:50 ratio with fructose (green), or C) mannose (orange). Lighter color indicates exponential points taken for growth rate calculations. Data were from seven independent biological replicates.

Table S2. Growth parameters[a] of *P. protegens* Pf-5.

Condition[b]	Growth Rate (hr^{-1})	Uptake Rate (mmol $gCDW^{-1}$ hr^{-1})	$Y_{x/s}$ (g/g)	Maximum concentration (mM)		
				Gluconate	2-Ketogluconate	Pyruvate
Glucose	0.56 ± 0.09	13.35 ± 3.28[c] (10.95 ± 2.24)[d]	0.39 ± 0.03	3.71 ± 0.16	2.34 ± 0.73	0.13 ± 0.08
Glucose:Fructose	0.52 ± 0.06	Glucose: 7.01 ± 1.70[c] (4.11 ± 1.69)[d]<br>Fructose: 2.02 ± 0.86	0.42 ± 0.11	3.33 ± 0.39	2.73 ± 0.68	0.028 ± 0.027
Glucose:Mannose	0.50 ± 0.04	Glucose: 7.21 ± 2.74[c] (5.70 ± 2.13)[d]<br>Mannose: 1.91 ± 1.11	0.40 ± 0.11	2.16 ± 0.47	0.36 ± 0.14	0.077 ± 0.019

[a]The values in the table represent means ± standard deviation. Data were calculated from three independent biological replicate (*n* = 3), except for growth rate where *n* = 7.
[b]The total caron-equivalent concentration of the growth substrates was 100 mM C (or 3 g L^{-1}).
[c]Uptake rate of glucose into the periplasm
[d] Uptake rate modified to account for secretions of gluconate and 2-ketogluconate.

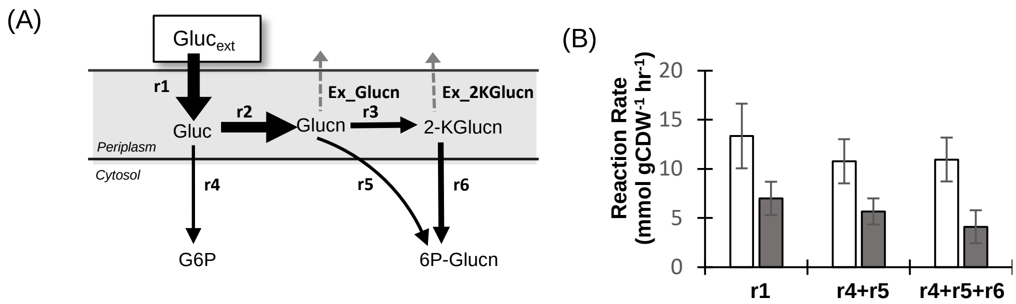

Fig. S2. Constraints in the MFA to account for secretions of gluconate and 2-ketogluconate from the periplasm.
(A) Schematic of periplasmic reactions and (B) calculated rates comparing during growth on glucose only (white bars) or on equimolar glucose and fructose (gray bars). Error bars represent 95% confidence intervals. Data were obtained from three independent biological replicates.

Table S3. Intracellular metabolic rates determined from quantitative flux modeling of the metabolism of [1,2-$^{13}C_2$]-glucose in *P. protegens* Pf-5 using the 13CFLUX2 software. These metabolic fluxes are illustrated in Fig. 4 in the main text. Refer to the legends of Fig. 1 and Fig. 3 in the main text for the abbreviations of the metabolite names.

**Reactions**	**[1,2-$^{13}C_2$]-glucose** *mmol gCDW^{-1} h^{-1}*
$Gluc_{ext}$ -> $Gluc_{peri}$	13.35 ± 3.28
$Gluc_{peri}$ -> G6P	0.64 ± 0.45
$Gluc_{peri}$ -> $Glucn_{peri}$	12.71 ± 0.45
$Glucn_{peri}$-> 6P-Glucn	7.69 ± 0.68
$Glucn_{peri}$ -> 2-$ketoglucn_{peri}$	1.70 ± 0.86
2-$ketoglucn_{peri}$ -> 6P-Glucn	0.16 ± 0.14
G6P -> 6P-Glucn	1.19 ± 0.58
6P-Glucn -> Pyr + GAP	8.97 ± 0.08
GAP -> 3-PG	6.21 ± 0.72
3-PG -> PEP	5.40 ± 0.76
PEP -> Pyr	3.59 ± 0.47
Pyr -> AcCoA + CO2	10.03 ± 0.95
DHAP -> GAP	-1.22 ± 0.30
FBP -> DHAP + GAP	-1.16 ± 0.29
FBP -> F6P	1.16 ± 0.29
F6P -> G6P	0.63 ± 0.20
6P-Glucn -> Ru5P	0.06 ± 0.11
Ru5P-> R5P	0.59 ± 0.02
Ru5P -> Xu5P	-0.53 ± 0.09
Xu5P + R5P -> GAP + S7P	-0.15 ± 0.04
S7P + GAP -> E4P + F6P	-0.15 ± 0.04
E4P + Xu5P -> F6P + GAP	-0.39 ± 0.05
OAA + AcCoA -> Cit	10.03 ± 0.95
Cit -> αKG	10.03 ± 0.95
αKG -> Succ	9.36 ± 0.97
Succ -> Fum	9.36 ± 0.97
Fum -> Mal	9.49 ± 0.97
Mal -> Pyr + CO_2	8.47 ± 0.55
Pyr + CO_2 -> OAA	9.35 ± 0.44
PEP + CO_2 -> OAA	1.27 ± 0.34
OAA -> IMP + Fum	0.13 ± 0.001

Table S4. Biomass and excretion efflux rates determined from quantitative flux modeling of the metabolism of [1,2-$^{13}C_2$]-glucose in *P. protegens* Pf-5 using the13CFLUX2 software. These metabolic fluxes are illustrated in Fig. 4 in the main text. Refer to the legends of Fig. 1 and Fig. 3 in the main text for the abbreviations of the metabolite names.

**Reactions**	**[1,2-$^{13}C_2$]-glucose** *mmol gCDW^{-1} h^{-1}*
aKG -> Biomass	0.67 ± 0.02
DHAP -> Biomass	0.06 ± 0.004
E4P -> Biomass	0.24 ± 0.02
G6P -> Biomass	0.08 ± 0.01
OAA -> Biomass	1.48 ± 0.03
PEP -> Biomass	0.54 ± 0.03
3PG -> Biomass	0.81 ± 0.04
Pyruvate -> Biomass	1.65 ± 0.04
R5P -> Biomass	0.74 ± 0.02
$Glucn_{in}$ -> $Glucn_{ext}$	3.33 ± 0.72
2-$ketoglucn_{in}$ -> 2-$ketoglucn_{ext}$	1.54 ± 0.88

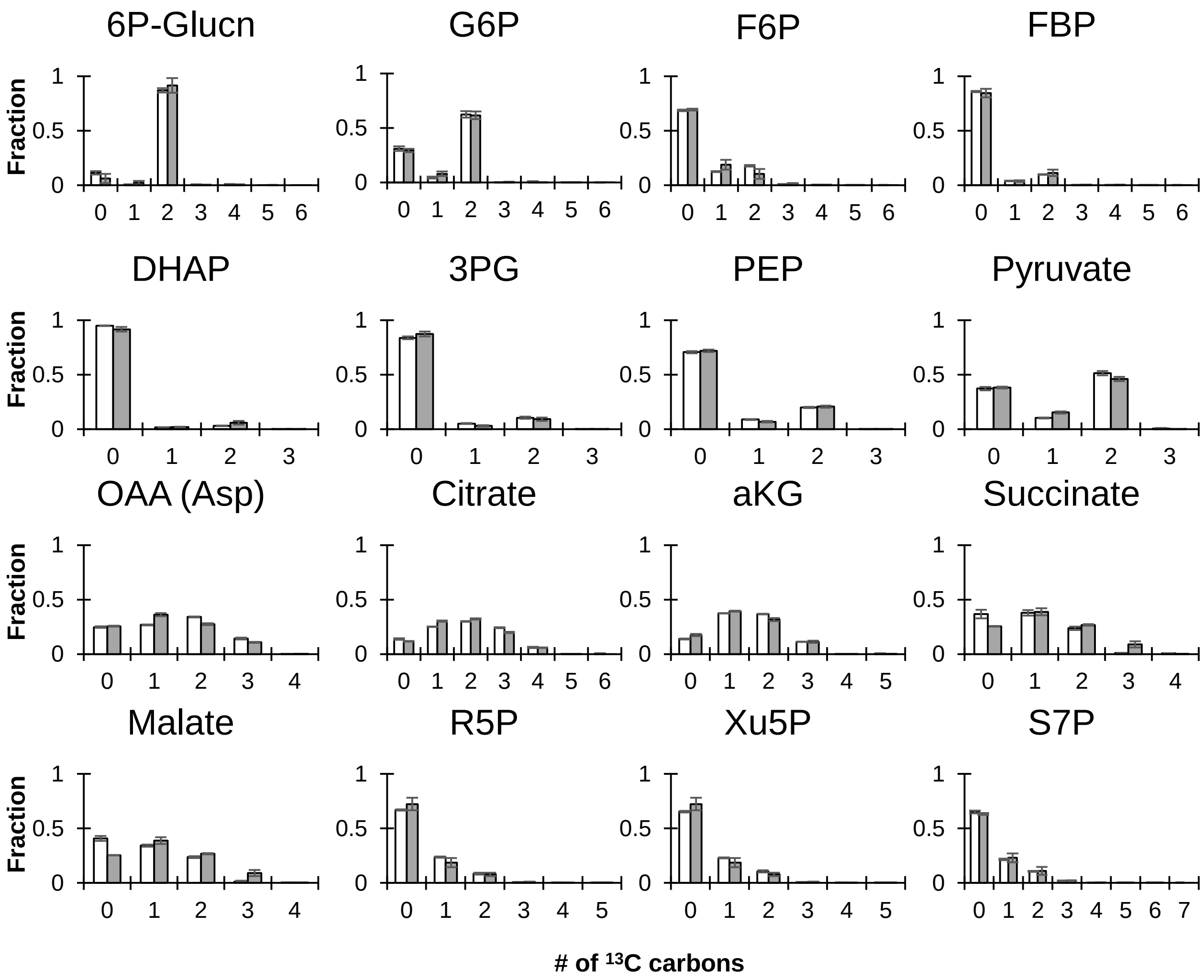

Fig. S3. Experimentally-determined (white bars) and model-estimated (gray bars) isotopomer distributions in the metabolite labeling patterns during *P. protegens* Pf-5 growth on [1,2-13C2]-glucose. Data (average ± standard deviation) were from optimizations of experimental data obtained from three independent biological replicates averaged across two timepoints (OD_{600} of 0.5 and OD_{600} of 1).

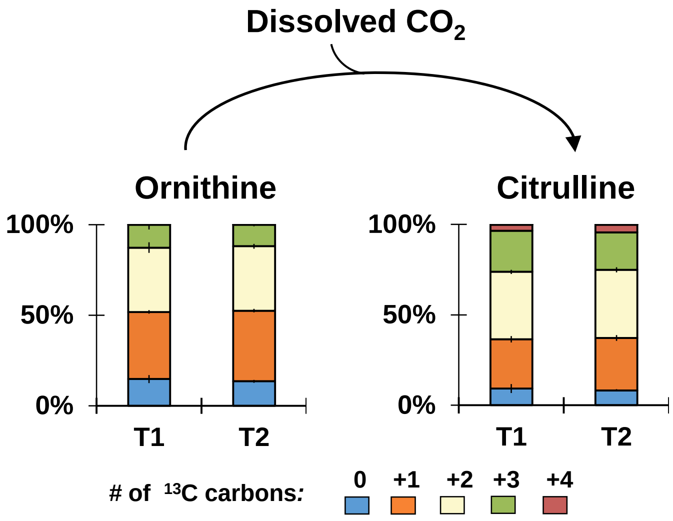

Fig. S4. Estimation of CO_2 labeling from the biosynthesis of citrulline from ornithine. Citrulline is formed from ornithine by the incorporation of dissolved CO_2. Addition of ^{13}C-labeled carbons in citrulline is taken as addition of labeled dissolved CO_2.

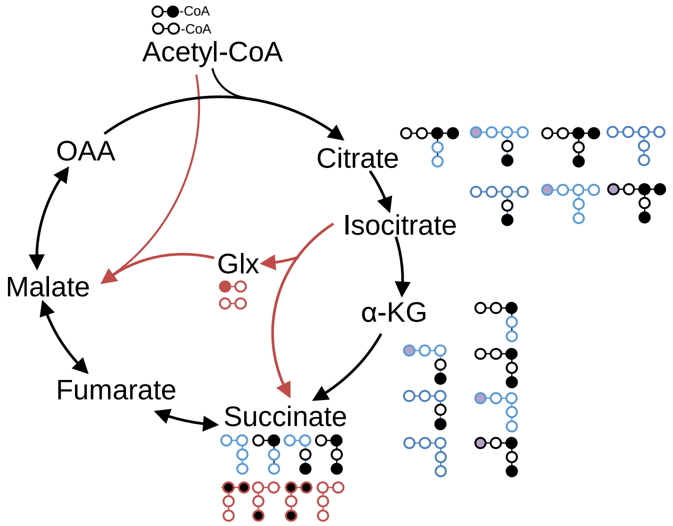

Fig. S5. Carbon mapping of metabolite labeling in the traditional TCA cycle and the glyoxylate shunt. Filled and empty and white circles indicate, respectively, ^{13}C-labeled and non-labeled carbons. Red-lines represent metabolites generated from the glyoxylate shunt; purple-filled circles represent incorporation of labeled carbon dioxide (CO_2) in solution. Oxaloacetate, OAA.

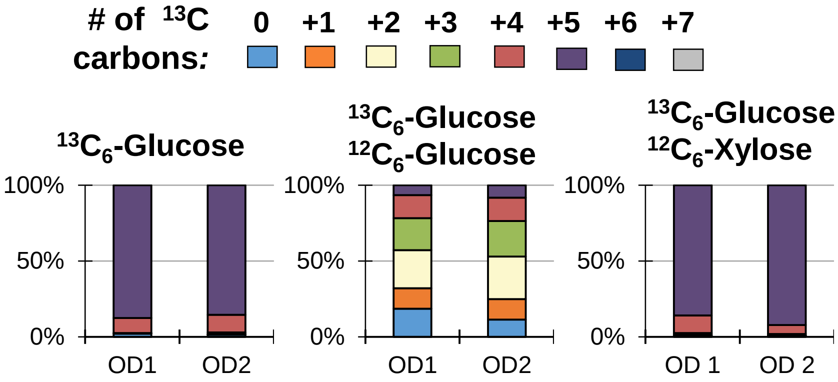

Fig. S6. Metabolite labeling pattern of α-ketoglutarate during growth on [U-$^{13}C_6$]-glucose ($^{13}C_6$-Glucose) alone or with unlabeled glucose ($^{12}C_6$-Glucose) or unlabeled xylose ($^{12}C_6$-Xylose).

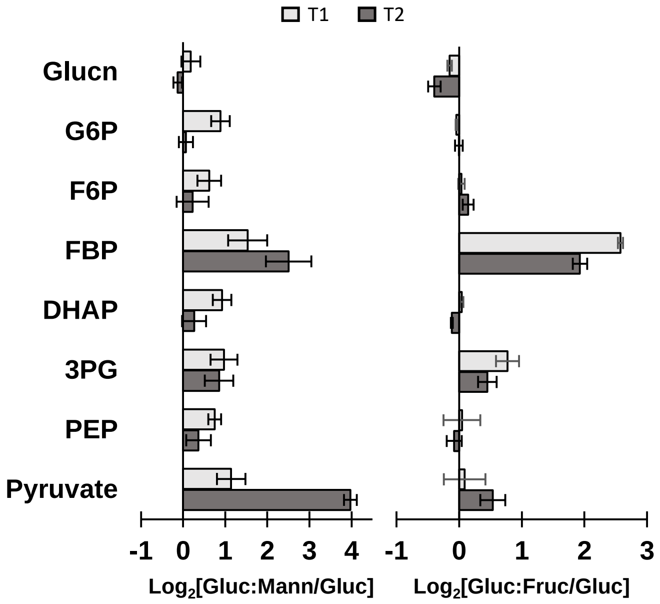

Fig. S7. Pool sizes of gluconate (Glucn) and metabolites in the EMP pathway. Data (average ± standard deviation) were from three independent biological replicates.

Table S5. Intracellular metabolic rates determined from quantitative flux modeling of the metabolism of ^{13}C-labeled glucose (UGluc) with unlabeled glucose (Gluc) or fructose (Fruc) in *P. protegens* Pf-5 using the13CFLUX2 software. These metabolic fluxes are illustrated in Fig. 7 in the main text. Refer to the legends of Fig 1 and Fig. 3 in the main text for the abbreviations of the metabolite names.

**Reactions**	**UGluc:Gluc**	**UGluc:Fruc**
	mmol gCDW^{-1} h^{-1}	
$Gluc_{ext}$ -> $Gluc_{peri}$	13.35 ± 3.28	7.01 ± 1.70
$Fruc_{ext}$ -> F1P	N/A	1.29 ± 0.05
F1P -> FBP	N/A	1.29 ± 0.05
$Gluc_{peri}$ -> G6P	1.19 ± 0.24	0.85 ± 0.14
$Gluc_{peri}$ -> $Glucn_{peri}$	12.16 ± 0.24	6.16 ± 0.14
$Glucn_{peri}$-> 6P-Glucn	7.80 ± 0.35	3.79 ± 0.14
$Glucn_{peri}$ -> 2-$ketoglucn_{peri}$	1.68 ± 0.71	1.17 ± 0.16
2-$ketoglucn_{peri}$ -> 6P-Glucn	1.02 ± 0.45	0.69 ± 0.14
G6P -> 6P-Glucn	1.97 ± 0.12	2.39 ± 0.21
6P-Glucn -> Pyr + GAP	10.71 ± 0.63	6.68 ± 0.24
GAP -> 3-PG	7.86 ± 0.77	4.84 ± 0.14
3-PG -> PEP	7.19 ± 0.76	4.11 ± 0.14
PEP -> Pyr	6.75 ± 0.76	3.63 ± 0.14
DHAP -> GAP	-1.30 ± 0.13	-0.79 ± 0.05
FBP -> DHAP + GAP	-1.25 ± 0.13	-0.73 ± 0.05
FBP -> F6P	1.25 ± 0.13	2.03 ± 0.11
F6P -> G6P	0.85 ± 0.13	1.61 ± 0.13
6P-Glucn -> Ru5P	0.08 ± 0.02	0.18 ± 0.10
Ru5P -> R5P	0.48 ± 0.04	0.60 ± 0.03
Ru5P -> Xu5P	-0.40 ± 0.03	-0.42 ± 0.04
Xu5P + R5P -> GAP + S7P	-0.10 ± 0.02	-0.10 ± 0.03
S7P + GAP -> E4P + F6P	-0.10 ± 0.02	-0.10 ± 0.03
E4P + Xu5P -> F6P + GAP	-0.30 ± 0.01	-0.32 ± 0.01

Table S6. Biomass and excretion efflux rates of metabolites determined from quantitative flux modeling of the metabolism of ^{13}C-labeled glucose (UGluc) with unlabeled glucose (Gluc) or fructose (Fruc) by *P. protegens* Pf-5 using the13CFLUX2 software. These metabolic fluxes are illustrated in Fig. 7 in the main text. Refer to the legends of Fig 1 and Fig. 3 in the main text for the abbreviations of the metabolite names.

**Reactions**	**UGluc:Gluc**	**UGluc:Fruc**
	mmol gCDW^{-1} h^{-1}	
DHAP -> Biomass	0.051 ± 0.001	0.057 ± 0.001
E4P -> Biomass	0.21 ± 0.01	0.23 ± 0.01
G6P -> Biomass	0.064 ± 0.000	0.071 ± 0.001
PEP -> Biomass	0.441 ± 0.001	0.49 ± 0.003
3PG -> Biomass	0.67 ± 0.01	0.74 ± 0.01
Pyruvate -> Biomass	1.37 ± 0.08	1.48 ± 0.04
R5P -> Biomass	0.57 ± 0.06	0.65 ± 0.04
$Glucn_{in}$ -> $Glucn_{ext}$	2.69 ± 0.92	1.46 ± 0.12
2-$ketoglucn_{in}$ -> 2-$ketoglucn_{ext}$	0.66 ± 0.26	0.37 ± 0.03

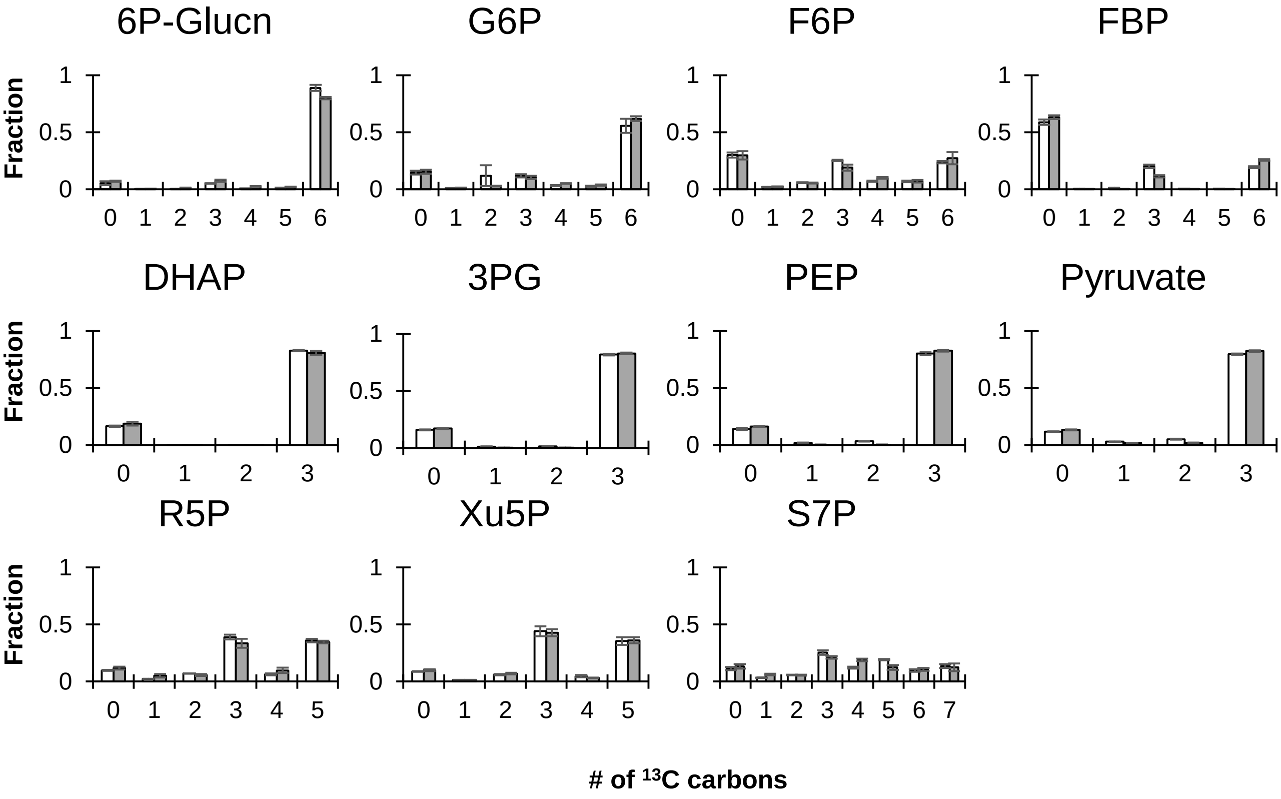

Fig. S8. Experimentally-determined (white bars) and model-estimated (gray bars) isotopomer distributions in the metabolite labeling patterns during *P. protegens* Pf-5 growth on 50:50 [$U^{13}C_6$]-glucose and unlabeled fructose. Data (average ± standard deviation) were from optimizations of experimental data obtained from three independent biological replicates (OD_{600} of 0.5-0.6).

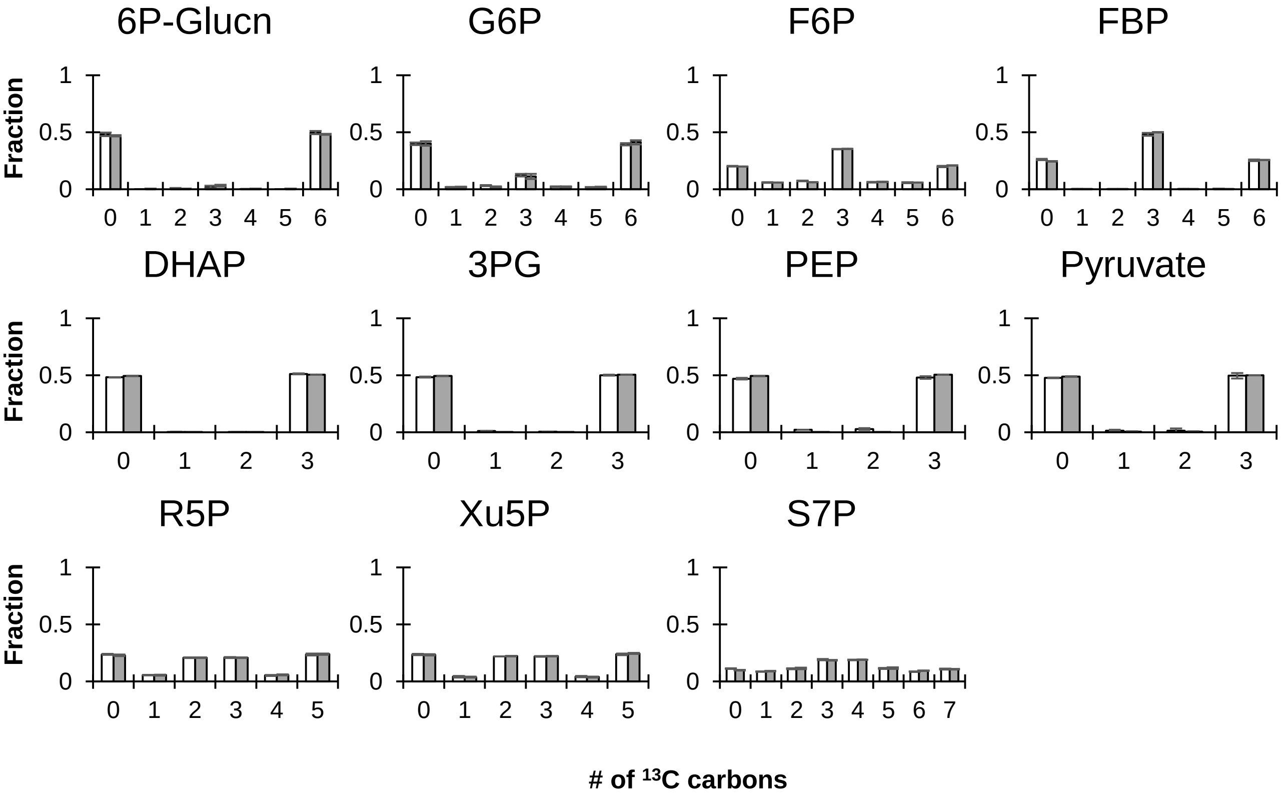

Fig. S9. Experimentally-determined (white bars) and model-estimated (gray bars) of isotopomer distributions in the carbon labeling patterns of *P. protegens* Pf-5 grown on 50:50 [$U^{13}C_6$]-glucose and unlabeled glucose. Data presented were the average of three independent optimizations of experimental data of three separate bioreplicates (OD_{600} of 0.5-0.6). Error bars represent standard deviation.

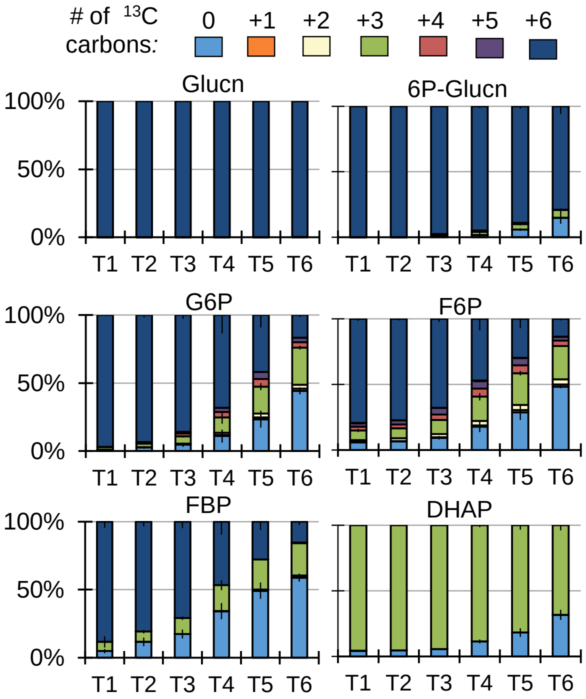

Fig. S10. Kinetic profiling of metabolite labeling patterns in cells grown simultaneously on [U-$^{13}C_6$]-glucose and unlabeled mannose. Color legend: nonlabeled carbon (light blue), one ^{13}C-carbon (orange), two ^{13}C-carbons (cream), three ^{13}C-carbons (green), four ^{13}C-carbons (red), five ^{13}C-carbons (purple), six ^{13}C-carbons (dark blue). Data were obtained at six timepoints during exponential growth: at OD_{600} of 0.21-0.27 (T1), at OD_{600} of 0.40-.44 (T2), at OD_{600} of 0.54-0.67 (T3), at OD_{600} of 0.79-.87 (T4), at OD_{600} of 0.90-0.98 (T5), and at OD_{600} of 1.2-1.4 (T6). The data (average ± standard deviation) were from three independent biological replicates.

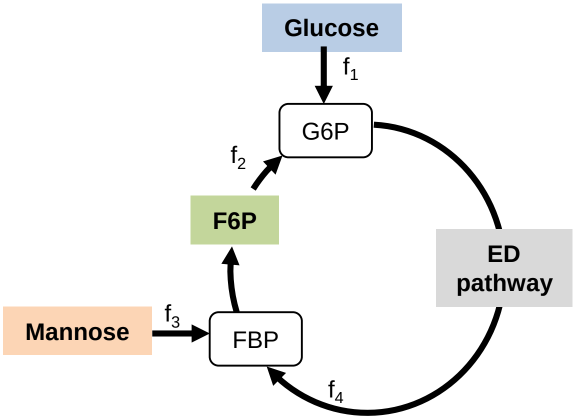

Equations for flux into G6P

$$G6P(0,3,6) = f_1(\text{Glucose}(0,3,6)) + f_2(\text{F6P}(0,3,6))$$

$$f_1 = \frac{G6P(0,3,6) - F6P(0,3,6)}{Glucose(0,3,6) - \text{F6P}(0,3,6)}$$

Equations for flux into FBP

$$FBP(0,6) = f_3(\text{Mannose}(0,6)) + f_4(\text{DHAP}(0,3))^2$$

$$f_3 = \frac{FBP(0,6) - DHAP(0,3)^2}{Mannose(0,6) - DHAP(0,3)^2}$$

Fig. S11. Network schematic and equations used for metabolic flux ratio analysis of the fractional flux into G6P from glucose (f_1) and F6P (f_2) or the fractional flux into FBP from mannose (f_3) and ED pathway (f_4) .
